# The Rules of Human T Cell Fate *in vivo*

**DOI:** 10.3389/fimmu.2020.00573

**Published:** 2020-04-08

**Authors:** Pedro Costa del Amo, Bisrat Debebe, Milad Razavi-Mohseni, Shinji Nakaoka, Andrew Worth, Diana Wallace, Peter Beverley, Derek Macallan, Becca Asquith

**Affiliations:** ^1^Department of Infectious Disease, Imperial College London, London, United Kingdom; ^2^Department of Biomedical Engineering, Johns Hopkins University, Baltimore, MD, United States; ^3^Precursory Research for Embryonic Science and Technology (PRESTO), Japan Science and Technology Agency, Kawaguchi, Japan; ^4^Faculty of Advanced Life Science, Hokkaido University, Sapporo, Japan; ^5^The Jenner Institute Laboratories, University of Oxford, Oxford, United Kingdom; ^6^Division of Infection and Immunity, University College London, London, United Kingdom; ^7^TB Research Centre, National Heart and Lung Research Institute, Imperial College London, London, United Kingdom; ^8^Institute for Infection and Immunity, St. George's Hospital, University of London, London, United Kingdom

**Keywords:** lymphocyte, fate, decision, proliferation, lifespan, mathematical model, labeling, half-life

## Abstract

The processes governing lymphocyte fate (division, differentiation, and death), are typically assumed to be independent of cell age. This assumption has been challenged by a series of elegant studies which clearly show that, for murine cells *in vitro*, lymphocyte fate is age-dependent and that younger cells (i.e., cells which have recently divided) are less likely to divide or die. Here we investigate whether the same rules determine human T cell fate *in vivo*. We combined data from *in vivo* stable isotope labeling in healthy humans with stochastic, agent-based mathematical modeling. We show firstly that the choice of model paradigm has a large impact on parameter estimates obtained using stable isotope labeling i.e., different models fitted to the same data can yield very different estimates of T cell lifespan. Secondly, we found no evidence in humans *in vivo* to support the model in which younger T cells are less likely to divide or die. This age-dependent model never provided the best description of isotope labeling; this was true for naïve and memory, CD4^+^ and CD8^+^ T cells. Furthermore, this age-dependent model also failed to predict an independent data set in which the link between division and death was explored using Annexin V and deuterated glucose. In contrast, the age-independent model provided the best description of both naïve and memory T cell dynamics and was also able to predict the independent dataset.

## Introduction

The relationship between cell division, differentiation, and death is key to many processes in adaptive immunity. Nowhere is this more fundamental to the immune response than in the case of lymphocyte populations, which mount rapid, dramatic expansions and contractions following pathogen challenge; generating both short-lived effectors and long-term memory populations, within the overall context of decades-long lymphocyte homeostasis. Mathematical models of lymphocyte dynamics typically assume that the processes that determine a lymphocyte's fate (division, differentiation, death) are stochastic and independent of the cell's age (1–7); where cell age is defined as the time since the cell arose from mitosis (Box 1). That is, the instantaneous probability of an event is constant and independent of age, hence the probability of a cell dying in the next hour is the same for a “newborn” cell as it is for a 20 year-old cell (provided they are drawn from the same population). Recently, the validity of the age-independent model has been challenged. In a series of elegant studies from the Hodgkin group (8–10), both population level and single cell analysis were used to investigate the interplay between lymphocyte fates. This was formulated into a conceptual age-dependent framework known as the “cyton” model (11). The central rules of the cyton model are that independent stochastic machinery governs cell processes (e.g., division, differentiation, death), that these processes are competitive and that this machinery is reset upon cell division with (to first approximation) no inheritance of lifespan. Thus, if a cell reaches its given time to divide before it reaches its time to die, its progeny will be preserved and indeed expand, whereas, if the converse applies, the cell's progeny will not survive. It is argued that variance in the timing of these events underlies essential features of the adaptive immune response such as rapid expansion or the decision between tolerance and activation. The cyton model has a number of distinct features. In particular cells have an intrinsic “age” which is reset on division; cells that have recently divided are therefore “younger” and have longer to live on average than cells which have not divided recently.

An alternative age-dependent framework, which we refer to as the “risk” model, has also been considered in the literature (12). According to the risk model, cells which have recently divided are more, not less, likely to die (13). For T lymphocytes the link between cell activation and cell death, activation-induced cell death, is well-recognized, but its contribution to steady state lymphocyte homeostasis is less clear (14).

Box 1 **Definition of terms.**

**Table:** 

Age of a cell	Time since cell was created by division (“born”) i.e., mitosis results in two daughter cells both of age 0 days
Lifespan of a cell	Time from creation of a cell by division to its loss by division, death or differentiation
Age-independent fate	The probability of a cell's death, differentiation or division is independent of its age
Birth of a cell	Creation of a cell by division of the mother cell

Which of these three paradigms (age-independent, cyton or risk) best describes lymphocyte fate is unknown. Age-independence, embodied in the exponential distribution (Supplementary Figure S1), is frequently assumed. At least part of its popularity is due to mathematical convenience: it is a minimal assumption model in which cellular behavior can be described by a single parameter represented as a rate in ordinary differential equations. However, its popularity is not just attributable to mathematical convenience, there is also a large body of data consistent with exponential survival or growth. Labeled lymphocytes in mice and humans disappear with an apparently constant probability *in vivo* (3, 15–17) and CD4^+^ T cell reconstitution following highly active antiretroviral therapy in HIV-1 infection appears to be exponential (18, 19). However, the age-independent model fails to explain a number of *in vitro* observations. Firstly, close examination of the time-to-die of naïve B cells shows an initial lag followed by an increasing rate of death, consistent with an age-dependent probability of dying. When IL-4 is added, the time to die of the entire population shifts; consistent with a change in the mean of a lognormally distributed time to die rather than a change in the mean of exponentially distributed times to die (10). Secondly, the addition of anti-CD3 to naïve CD4^+^ T cells shifts the time at which the cells enter their first division, consistent with a lognormally distributed time to first division (20). Thirdly, there is evidence for competition between cell death and cell proliferation as stimulation of naïve B cells with anti-CD40 and IL-4 had no impact on the loss rate of cells until the time at which surviving cells started to divide (10, 20). The same pattern has been reported for T cells stimulated with αCD3 (20); again this is not consistent with constant probabilities of proliferation and death. Finally, the cyton model produced good fits to both population level and single cell data from CFSE-stained, stimulated B and T cells (10, 11, 21).

These *in vitro* observations support an age-dependent cyton model in which recently divided cells have a reduced risk of death. Furthermore, the authors argue that the cyton model cannot be ruled out by the observation of exponential growth and survival curves since, for certain parameter combinations, age-dependent lognormally distributed times-to-proliferate and times-to-die generate growth and survival curves that are practically indistinguishable from exponential for most experimental set ups (22). The authors even describe parameter combinations for a single lognormal such that the resulting survival curve appeared biphasic—a common property of many data sets, typically taken as evidence for multiple (age-independent) subpopulations with different kinetic behavior (7, 12, 23). Consequently, appearance of exponential survival or growth is not sufficient evidence to rule out age-dependent processes. Hodgkin et al. have recently extended the cyton model by identifying the mechanism that controls the duration of the clonal burst undergone by B and T cells *in vitro* upon stimulation (24, 25). In the original version of the cyton model the duration of the proliferative burst (but not the proliferation rate) was inherited between generations but the underlying mechanism was unknown. Analysis of T and B lymphocytes post-stimulation under different conditions revealed that Myc expression levels were approximately the same in all cells upon the termination of the clonal burst, suggesting that Myc levels need to surpass a certain threshold for cell division to occur. Myc expression rates and consequently the duration of the proliferative burst (but again not the proliferation rates) were shown to be inherited with each division. This work supports and extends the cyton model.

In short, there is very good evidence to support the age-dependent cyton model of cell fate for both B and T lymphocytes. However, in order to have a system that can be finely manipulated and readily observed, work to date supporting the cyton model has been conducted almost entirely in murine cells *in vitro*. The one exception which we are aware of is an *in vivo* analysis of T cells in LCMV-infected mice [Supplementary Information in (10)]. However, in this work only bulk cell populations were studied, the analysis was restricted to mice and competing models were not considered. It therefore remains an open question whether the cyton model, risk model or age-independent model governs lymphocyte fate in humans *in vivo*.

Which model determines lymphocyte fate *in vivo* is a fundamental question with a number of consequences. Firstly, the assumption that lymphocytes are “age-less” underpins the interpretation of many experiments including labeling with stable isotopes, CFSE and BrdU as well as *in vivo* killing assays (4, 26–28). Changing this assumption could affect both the dynamic parameters estimated as well as the biological interpretation of the results (29). Given the ubiquitous application of these techniques (including the study of T cell depletion in HIV-1-infected individuals, T regulatory cell lifespan, T cell origin, cell dynamics in leukemia and diabetes) changes to their interpretation could have widespread impact (25). Secondly, immunity is a dynamic process. Understanding how lymphocytes quantitatively regulate their decision-making processes is essential for understanding basic lymphocyte biology.

Here we investigate the rules determining the fate of T lymphocytes in humans *in vivo* during healthy homeostasis. We consider CD4^+^ and CD8^+^ naïve and memory T cells and focus on two fates: death and division. Our approach is to combine stable isotope labeling *in vivo* (both with heavy water and deuterated glucose), Annexin V staining *ex vivo* and mathematical modeling. We aim to determine: (i) whether the choice of model makes a significant difference to estimates of parameters describing lymphocyte kinetics, (ii) which model best describes heavy water labeling patterns in naïve and memory T cells, and (iii) which model best predicts a new dataset which quantifies the relationship between short-term *in vivo* labeling with deuterated glucose and Annexin V binding as an indicator of incipient death. In this way we set out to elucidate the patterns of division and death which underlie normal lymphocyte behavior *in vivo*.

## Results

### Impact of Model Paradigm on Estimates of Lymphocyte Kinetics *in vivo*

Stable isotope labeling is an established technique for quantifying cell dynamics (5, 30, 31). Briefly, subjects are given deuterium in the form of deuterated glucose or heavy water. Deuterium is incorporated into the DNA of cells when they divide and lost when labeled cells die or differentiate. Cells of interest are sampled and the level of deuterium incorporated into their DNA is quantified by mass spectrometry. The rate of cell proliferation and the rate of cell disappearance are estimated by fitting a mathematical model to the labeling data and finding the values of the rates which minimize the discrepancy between data and prediction. The mathematical models used to interpret stable isotope labeling data typically assume that cells proliferate and die/differentiate in an age-independent manner (3, 5, 7, 23, 32–37). To investigate the extent to which estimates of proliferation would change if the age-independent assumption was relaxed, we developed an agent-based model (ABM) of a population of cells in homeostasis (Methods). The ABM simulated the labeling and delabeling phases of a deuterium labeling experiment, modeling division and disappearance events according to each of the paradigms of cell fate. In the ABM, cells were initialized upon birth (mitosis) with times to divide and to disappear drawn from a gamma or lognormal distribution. We investigated three paradigms:

- the cyton model, in which the probability of a cell dying or dividing increases with its age;
- the risk model, in which the probability of death or division decrease as the cell ages;
- the age-independent model, in which the probability of death or division is independent of cell age.

The parameters of the distribution determine which paradigm the simulated population obeys (Supplementary Figure S1). When the distribution is exponential (gamma with rate = 1) then the probability of an event (conditioned on survival to that point) is independent of age and the age-independent model is captured. When the distribution is gamma with shape parameter lower than one then the distribution of time to an event has a zero mode and a steeper slope than the exponential distribution and the probability of an event decreases with age, i.e., the risk paradigm is captured. When the distribution is gamma with a shape parameter higher than one then the mode is greater than zero, probability of an event increases with age and the cyton paradigm is captured. For the lognormal distribution, the mapping between the parameters and paradigms is less precise but when the location parameter is large relative to the scale then the distribution is strongly right-skewed and the mode is much greater than zero (cells much less likely to die or divide immediately after a previous division event) and the cell behavior conforms to the cyton paradigm; as the location parameter decreases relative to the scale then the mode decreases and the behavior becomes risk-like. We reiterate that we define the age of a cell as the time since creation of the cell by mitosis (Box 1).

We considered both a homogeneous cell population and a heterogeneous population consisting of two subpopulations. Under the heterogeneous scenario, both subpopulations were governed by the same form of distribution (i.e., two exponentials, two gammas or two lognormals) with varying parameters. We thus had 6 models (Table 1): two (Model 1 and 2) were age-independent, the remaining four were age-dependent models with Models 3 and 4 obeying the cyton paradigm and models 5 and 6 the risk paradigm. For the cyton and risk models we considered the populations to be governed either by gamma or lognormal distributions; for brevity we focus on results from the gamma distribution in the text; results obtained with the lognormal distribution are similar unless stated otherwise and are presented in the figures and Supplementary Information.

**Table 1:** Summary of the different models considered.

**Model**	**Paradigm**	**Number of subpopulations**	**Distribution of time to die and divide**	**Number of free parameters**
Age-independent, homogeneous	Age-independent	1	Exponential (gamma with shape = 1)	1
Age-independent, heterogeneous	Age-dependent	2	Exponential (gamma with shape = 1)	3
Cyton, homogeneous	Cyton	1	Gamma or lognormal	2
Cyton, heterogeneous	Cyton	2	Gamma or lognormal	5
Risk, homogeneous	Risk	1	Gamma or lognormal	2
Risk, heterogeneous	Risk	2	Gamma or lognormal	5

*The number of kinetically distinct subpopulations, type of distribution for times to die/differentiate and to divide, and number of free parameters for each of the 6 models. We considered 3 paradigms (age-independent, cyton, risk); 6 models (i.e., homogeneous and heterogeneous versions of each paradigm) with 10 formulations in total (as each of the age-dependent models can be described either by the gamma or the lognormal distribution)*.

These models, representing the three paradigms, were fitted to experimental data from a published stable isotope-labeling study (32). In this study, 5 healthy human volunteers were given heavy water (^2^H_2_O) for 63 days and the fraction of label incorporated in the DNA of naïve (CD27^−^CD45RO^−^) and memory (CD45RO^+^) CD4^+^ and CD8^+^ T cells was measured at successive time points.

#### Naïve CD4^+^ and CD8^+^ T Cells

**Figure 1:**
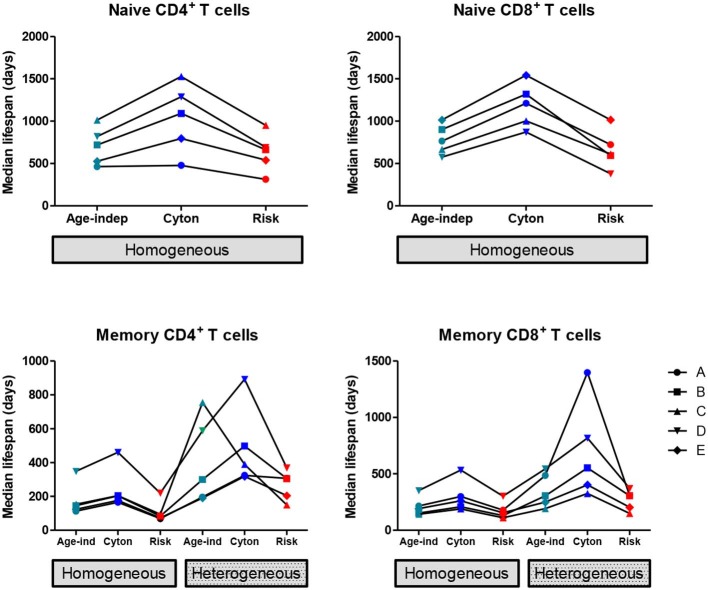
Impact of model assumptions on lifespan estimates. Median lifespan estimates obtained by fitting models with different assumptions about cell fate to heavy water labeling data (32). Top row: naïve T cells, bottom row: memory T cells. Each line and symbol corresponds to a different subject (A–E). Here the cyton and risk models are described using the gamma distribution, similar results are obtained using the lognormal distribution. We use the color scheme age-independent: aqua, cyton: cobalt, risk: red. The lifespan of a cell is defined as the time to first event (division or death).

We found that the homogeneous population models consistently provided a better description of the naïve cell labeling data than heterogeneous population models, in agreement with the literature (7, 12). So, when considering the impact of model choice on parameter estimates, we restrict ourselves to the homogeneous versions of the three paradigms (age-independent, cyton, and risk). The median lifespan (time to death or division) estimated from the three models are shown in Figure 1; the distribution of lifespans are shown in Supplementary Figure S2. The age-dependent cyton model gave the longest estimates of naïve cell lifespans; they were systematically greater than lifespans obtained using the age-independent model (*P* = 0.002, mean difference = 366 days, 49% increase, Wilcoxon signed rank two-tailed) and even longer than lifespans obtained using the risk model (*P* = 0.002, mean difference = 465 days, 72% increase). The lognormal version of the cyton model gave similar results to the version based on the gamma distribution described above whilst the lognormal version of the risk model failed to fit the experimental data.

#### Memory CD4^+^ and CD8^+^ T Cells

Previous studies assuming age-independent models of lymphocyte dynamics have suggested that memory cell pools are kinetically heterogeneous—at least two exponentials being required to capture their dynamics (7, 38, 39). However, Dowling et al. (22) have shown that, for certain parameter combinations, the lognormal distribution results in biphasic survival curves which could be mistaken for the signature of a heterogeneous population, when age-dependence is not taken into account. Therefore, we do not make any assumption about whether the memory T cell populations are kinetically homogeneous or heterogeneous.

The estimated median lifespans are presented in Figure 1, the lifespan distributions are given in Supplementary Figure S3. In all cases the heterogeneous version of a model yielded longer lifespans than the corresponding homogeneous version (*P* = 2 × 10^−9^, mean difference = 212 days, 105% increase). Within both the homogeneous and heterogeneous models we saw the same ranking of lifespans as for naïve cells i.e., the cyton model gave the longest lifespans, followed by the age-independent model with the risk model giving the shortest lifespans. If the cyton model described by the lognormal distribution is mistakenly identified as heterogeneous age-independent, as suggested (22), then this would result in a significant overestimate of median lifespan (*P* = 0.002, mean difference in lifespan homogeneous cyton v heterogeneous age-independent = 298 days, 360% increase).

We conclude that assumptions about the underlying paradigm can have considerable impact on estimates of lymphocyte lifespan calculated from stable isotope labeling data.

### Which Paradigm Provides the Best Description of Stable Isotope Labeling Data?

#### Naïve CD4^+^ and CD8^+^ T Cells

**Figure 2:**
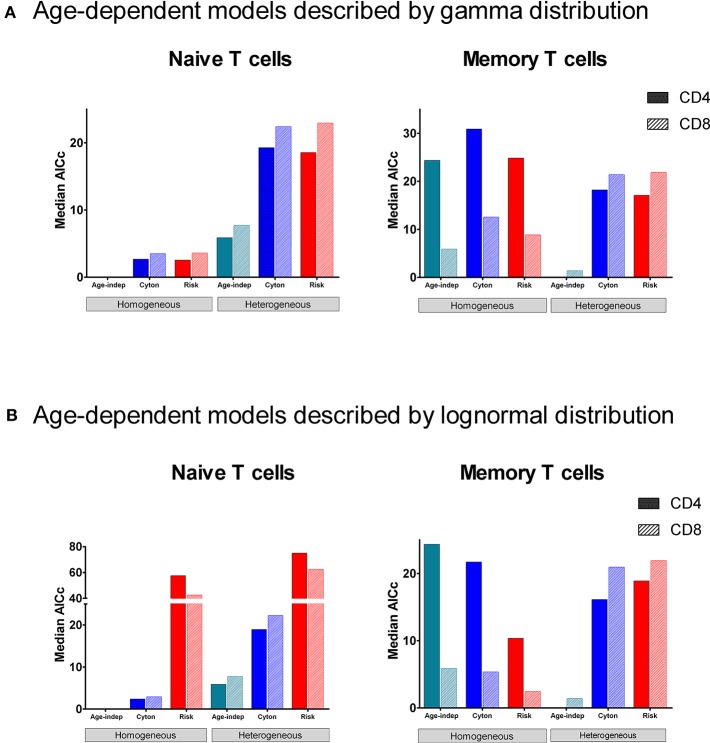
Median normalized AICc for the fit of each of the models to data from naïve and memory CD4^+^ and CD8^+^ T cells. The model with the lowest AICc is the best performing model. The age-independent model performs best both for naïve CD4^+^ and CD8^+^ T cells (homogeneous version) and for memory CD4^+^ and CD8^+^ T cells (heterogeneous version). The data fitted is from the heavy water study. **(A)** shows median normalized AICc for the gamma versions of the age-dependent models; **(B)** shows median normalized AICc for the lognormal versions of the age-dependent models. The individual AICc values are in Supplementary Tables S1, S2. We use the color scheme age-independent: aqua, cyton: cobalt, risk: red; solid color in bars denotes CD4^+^ cells, hatched color denotes CD8^+^ cells. Note the y axis scales are different in each panel (and broken in one panel). Absolute value of the AICc does not carry any meaning so we plot the normalized AICc which is the difference between the AICc of the model being investigated and the winning model (the normalized AICc is therefore zero for the winning model).

In order to determine which paradigm provides the best description of T cell fate we fitted the different models to the stable isotope labeling data described above and compared the quality of the fit using the Corrected Akaike Information Criterion [AICc (40), Methods]. For naïve CD4^+^ and CD8^+^ T cells, the homogeneous age-independent model provided a better description of the experimental data than either the homogeneous cyton or the homogeneous risk models in 10/10 cases (Figure 2, AICc for the age-independent model significantly lower than the best fitting age-dependent model *P* = 0.002, two tailed Wilcoxon, lower AICc indicating a better description) but the median difference in AICc was small (2.7). The heterogeneous cyton and risk models were not competitive (lose in 10/10 cases, *P* = 0.002) and the median difference in AICcs was large (19.5). The heterogeneous models failed because they have a larger number of parameters but do not offer a better fit than the homogeneous models (Supplementary Figure S4, Supplementary Table S1). In summary, for naïve CD4^+^ and CD8^+^ T cells, the data (i) support a homogeneous model rather than a heterogeneous model and (ii) provide no evidence to support age-dependence.

#### Memory CD4^+^ and CD8^+^ T Cells

**Figure 3:**
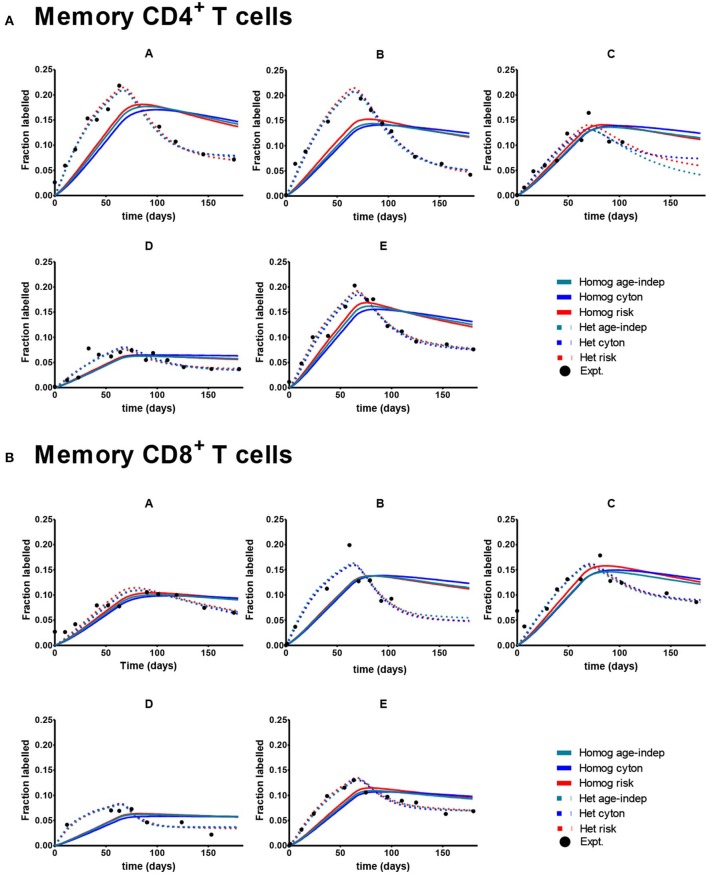
Best fit of the 6 models to Memory CD8^+^ and CD4^+^ T cell data. A different subject (A-E) is shown in each panel. Here the cyton and risk models are described using the gamma distribution; similar results are obtained using the lognormal distribution. We use the color scheme age-independent: aqua, cyton: cobalt, risk: red; solid lines denote best fit of homogeneous models, dashed lines best fit of heterogeneous models; black circles denote experimental data. **(A)** Depicts memory CD4^+^ T cells. **(B)** Depicts memory CD8^+^ T cells. Data taken from Vrisekoop et al. (32).

Despite the fact that we considered 4 times more age–dependent than age–independent models (risk and cyton; lognormal and gamma formulations) the age-independent models provided the best description of the memory T cell data in 7/10 cases. Where data was dense the heterogeneous version of the age-independent model was successful (5/10 cases); where data was limited the homogeneous age-independent model was preferred (Figure 2). The homogeneous risk model (lognormal distribution) was better in the remaining three cases, but by small differences in the AICc (mean 2.1, Supplementary Table S2). The cyton model (in either heterogeneous or homogeneous form) was never competitive; losing in 10/10 cases (mean difference in AICc between cyton and winning model: 22.1). The plots of the fits for Memory CD4^+^ and CD8^+^ T cells are shown in Figure 3. In summary, the dynamics of CD4^+^ and CD8^+^ T cell memory cells *in vivo* are best described by age-independent models; the cyton model performs poorly.

#### Approach Validation

**Figure 4:**
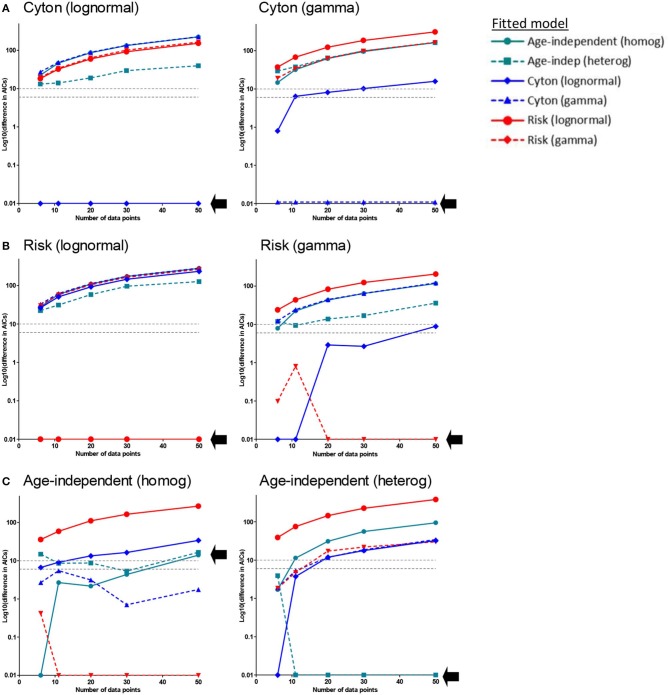
Approach Validation. *In silico* data (with noise) was generated using the paradigm named above each panel. Datasets of different sizes (*n* = 6, *n* = 11, *n* = 20, *n* = 30, *n* = 50) were constructed; *n* = 11 corresponds to the typical size of experimental data set analyzed in the previous section. We then used our approach to identify the generating model. Briefly, each of the models was fitted to the data and the normalized AICc is plotted. The normalized AICc for the winning model (which is zero by definition) is plotted as 0.01 since log_10_(0) is not defined. In each panel a different model was used to generate the data (generating model is named above each panel), each line represents the normalized AICc from fitting a different model to the generated data, the x axis is the size of the data set; the AICc of the generating model is indicated by a black arrow. The model with the lowest normalized AICc is the model identified as the generating model by our approach. **(A)** Generating model is the cyton model (either lognormal or gamma). The generating model was always correctly identified (lowest AICc) and, with the exception of one model for the smallest dataset (*n* = 6), no other model came within 6 AICc of the generating model **(B)** Generating model is the risk model. For the lognormal version the generating model was always correctly identified; for the gamma version the generating model was correctly identified the majority of the time and when it was not the winning model it was always within 1 AICc of the winning model (i.e., it was indistinguishable from the winning model). **(C)** Generating model is the age-independent model. For the age-independent heterogeneous model the winning model was the generating model for *n* ≥ 11, though for *n* = 11 (but not higher) other models were competitive. For the age-independent homogeneous model the winning model for most values of *n* was, unexpectedly, gamma risk. The gray dashed horizontal lines correspond to a difference in AICc of 6 and 10, these are the rule of thumb cut-offs typically used with the AICc. A difference of 6 is considered sufficient to support the winning model, a difference of 10 allows the favoring of the winning model with confidence.

It is noticeable that, when comparing the fit of the different paradigms to the experimental data, many models produce a similar fit and that the age-independent model therefore tends to win because it has fewer parameters. We were concerned that this approach may be biased against the more complicated models because the data may not be sufficiently dense to identify the age-dependent models. We therefore investigated whether age-dependent models, if they were true, would be expected to generate sufficient signal in these types of data sets to be identified. We generated *in silico* datasets of different sizes (with noise) consistent with a known paradigm and then investigated if our approach could correctly identify the generating paradigm (**Methods**). We found that when the generating model was the cyton model (either lognormal or gamma) the generating model was always correctly identified and (with the exception of one model for the smallest dataset n=6) no other model came within 6 AICc of the generating model (Figure 4A). When the generating model was risk, for the lognormal version the generating model was always correctly identified; for the gamma version the generating model was correctly identified the majority of the time and when it was not the winning model it was always within 1 AICc of the winning model i.e., it was indistinguishable from the winning model (Figure 4B). When the generating model was the age-independent heterogeneous model then the winning model was the generating model for *n* ≥ 11, though for *n* = 11 (but not higher) other models were competitive. For the age-independent homogeneous model then the winning model was, unexpectedly, gamma risk and this did not improve even for very large data sets (Figure 4C). On investigating this further, we found that the winning version of the gamma risk model always had shape parameter close to 1 (mean 0.97) i.e., the winning model was very close to the age-independent exponential but the additional freedom to slightly decrease the shape parameter resulted in an improved fit. Of note, there was never a case when data generated by any age-dependent paradigm was misclassified as age-independent. In summary, the approach performs well but has a slight bias against the age-independent model; it robustly identified the cyton model whenever it was the generating model. We conclude that the age-dependent models should be distinguishable from age-independent models with this type and density of data and that it is unlikely that cell dynamics consistent with the cyton model would be misidentified as coming from an age-independent model.

#### Alternative Approach

We have shown that fitting the models to the experimental data and comparing their AICcs provides support for the age-independent family of models. Furthermore, *in silico* testing indicated that this approach performs well. Nevertheless, we were still concerned that this approach suffers from a dependence on the number of data points relative to model complexity; we therefore implemented an alternative method. In the previous approach (above) we compared models on the basis of their AICcs by restricting parameter ranges such that the distributions obeyed either the age-independent, risk or the cyton paradigm. For the alternative approach, we took advantage of the fact that a single gamma distribution can describe all three paradigms. We allowed parameters to vary freely, effectively ranging over all paradigms, and then investigated which paradigm the best fit parameters corresponded to. In this way all models were considered equally without reference to model complexity. We reiterate that, for the gamma distribution a shape parameter <1 corresponds to the risk paradigm, a shape parameter equal to 1 corresponds to the age-independent paradigm and a shape parameter >1 corresponds to the cyton paradigm.

**Figure 5:**
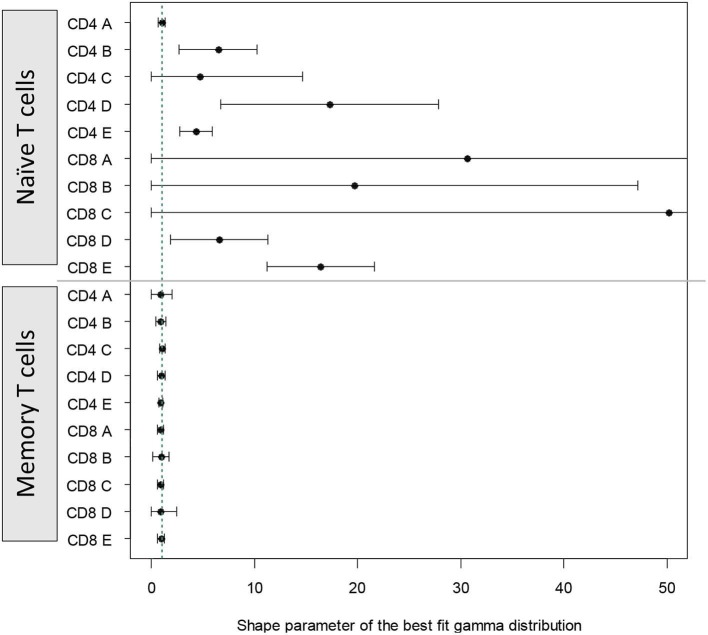
Alternative approach: shape parameter of the best fit gamma distribution. We fitted a model in which time to death/differentiation and time to division were drawn from gamma distributions with unconstrained shape and scale parameters. The model thus has the freedom to take up the risk paradigm (shape <1), the age-independent paradigm (shape = 1) or the cyton paradigm (shape > 1). The shape parameter of the best fit distributions to each of the cell populations is plotted above. Vertical green dashed line corresponds to shape = 1. The first 10 estimates (above the horizontal line) are for naïve T cells, the next 10 estimates (below the horizontal line) are for memory T cells.

For naïve T cells the shape parameter was poorly identifiable; in most cases the CI included 1 (i.e., shape parameter not significantly different from one). For memory T cells the shape parameter could be estimated more accurately and in all cases was very close to one (Figure 5). These results closely mirror those obtained using the AICc. That is, for naïve T cells it is difficult to distinguish between paradigms but there is no clear support for the cyton model. Whilst for memory T cells, the dynamics are best described by the age-independent paradigm.

We wished to extend this alternative approach to investigate the lognormal family of distributions. The problem arises that for the lognormal distribution, unlike the gamma distribution, there are no parameter combinations such that the lognormal distribution is age-independent, nor is there a precise mapping between parameter combinations and the age-dependent paradigms. Instead we looked at the (instantaneous) probability of death conditioned on survival to that point. If the underlying paradigm is risk then we would expect a strong left skewed distribution (i.e., cells more likely to die immediately after division) whereas if the underlying paradigm is cyton-like then then the probability of death would increase with time (older cells more likely to die). We found that for naïve T cells the distributions were rather poorly identified. For memory T cells the picture was much clearer, for both a homogenous and a heterogeneous lognormal model the probability distributions were much more consistent with the risk rather than the cyton paradigm (Supplementary Figure S5). Age independent paradigms could not be considered for the lognormal distributions within this framework but it is clear that there is little support for the cyton model.

### Measurement of Deuterated Glucose Labeling in Annexin V–Positive and –Negative Cells

A model's ability to predict new data is as important, and arguably a more stringent test, than its ability to fit existing data. We therefore generated an independent dataset consisting of a different type of data against which to assess the models. As we were interested in the temporal link between division and death we combined deuterated glucose pulse-labeling with separation of cells during follow-up according to a marker of incipient apoptosis, Annexin V binding. Annexin V is a protein which binds to phosphatidylserines translocated to the outer membrane during apoptosis; its binding on the cell surface is an early indicator of apoptosis. In this way we explored the relationship between recent division and risk of death.

**Figure 6:**
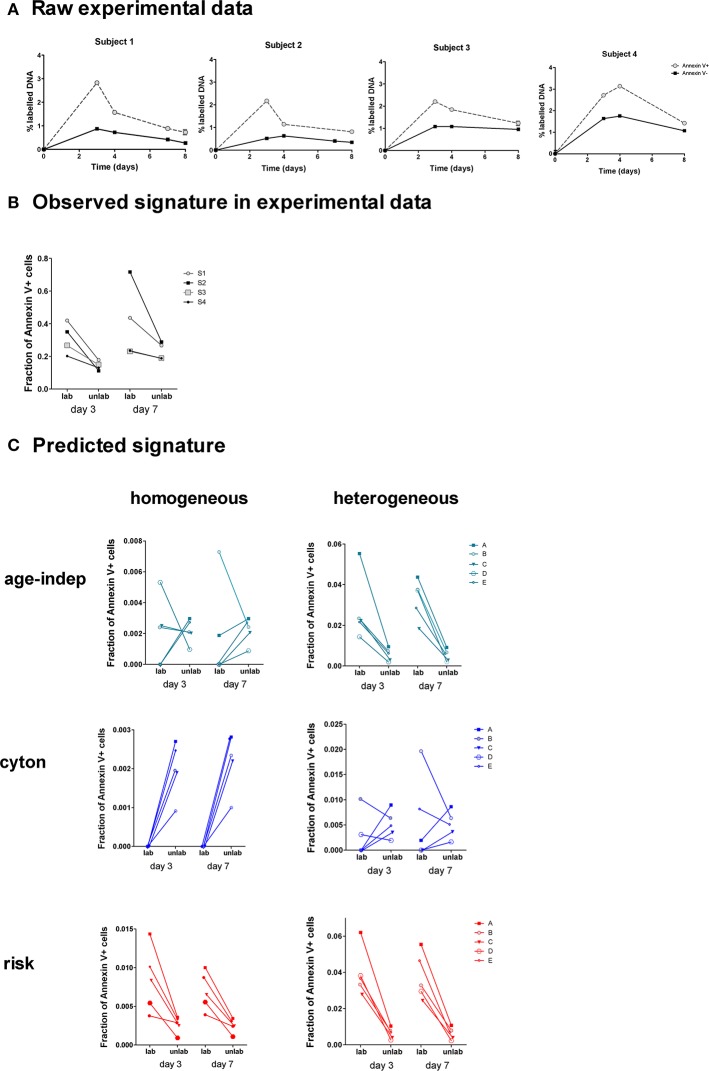
Observed and predicted signatures in Annexin V data set. **(A)** Four subjects were labeled with deuterated glucose for 24 h. The fraction of labeled DNA in Annexin V+ and Annexin V– CD4^+^CD45RO^+^ T cells was quantified (one subject per graph). **(B)** This was converted into a signature of the fraction of Annexin V+ cells amongst labeled and unlabeled cells. **(C)** We tested the ability of each of the 6 models to predict this signature. The heterogeneous age-independent and both homogeneous and heterogeneous risk models correctly predicted the signature; the homogeneous age-independent, homogeneous cyton and heterogeneous cyton models did not.

Four healthy adults received deuterated glucose ([6,6-^2^H_2_]-glucose) as an intravenous infusion for 24 h. Blood samples were taken at successive time points post-infusion and CD4^+^ memory (CD45RO^+^) T lymphocytes were sorted by flow cytometry into Annexin V-positive and Annexin V-negative subsets and deuterium incorporation into cellular DNA in each subset was quantified (Figure 6A).

### Prediction of Annexin V Labeling Signatures

We wished to ascertain the ratios of Annexin V-positive and -negative cells in labeled and unlabeled cell populations. This cannot be measured directly, i.e., we cannot sort labeled and unlabeled cells and then quantify Annexin V binding, as the measurement of label requires the destruction of the cells (their DNA is isolated and hydrolysed). Instead we calculate the ratio at days 3 and 7 from the proportion of label in the Annexin V-positive and -negative cells (**Methods**). This analysis revealed a distinct signature in the experimental data (Figure 6B): the fraction of Annexin V-binding cells in the labeled population was consistently higher than the fraction in the non-labeled population at both days 3 and 7 (*P* = 0.008, Wilcoxon two tailed). We investigated the ability of the 6 different models (Table 1) to predict this signature.

For each model we took the best fit parameter combinations generated in the previous section by fitting to heavy water labeling of Memory CD4^+^ T cells, and then used these parameters to predict the outcome of the Annexin V/deuterated glucose experiment using the ABM. Apoptosis has been shown to last for 12–24 h (41), and the translocation of phosphatidylserines to the outer part of the membrane of the cell is known to occur from an early stage of the process (42). Accordingly, we approximated the fraction of Annexin V-binding cells in labeled and non-labeled cell populations by the fraction of simulated cells with remaining lifetimes lower than 24 h. We then investigated the models' ability to predict the observed signatures. The fraction of Annexin V-binding cells *ex vivo* is known to overestimate the fraction of cells dying *in vivo* by at least 50-fold (43) [presumably because not all Annexin V-binding cells die (44, 45) and because taking cells *ex vivo* increases death (46)]; furthermore the individual subjects being simulated are different from the individuals subjects fitted so we did not aim to predict the absolute levels of label incorporation (which shows considerable inter-individual variation). Instead we aimed to predict the observed signature which was clear and consistent across all four individuals (Figure 6B).

Of the six models considered, three successfully predicted the observed signature. The heterogeneous age-independent, and both the homogeneous and heterogeneous risk models correctly predicted that the fraction of Annexin V+ cells was significantly higher amongst labeled cells than non-labeled cells at day 3 and at day 7 (Figure 6C). Intuitively, this makes sense: if the population is heterogeneous age-independent then the subpopulation with faster kinetics will be overrepresented amongst the labeled cells. These cells die faster than the slow population labeled cells and thus will express a higher proportion of Annexin V. Similarly, under the risk paradigm, recently divided (i.e., labeled) cells will have a greater risk of dying. The homogeneous age-independent model and both homogeneous and heterogeneous versions of the cyton model failed to predict the signature and were not consistent with the experimental observations (Figure 6C).

## Discussion

T cell proliferation and death are typically assumed to be independent of cell age (where age is defined as time since division). However, it has been postulated that lymphocyte division and death are governed by the age-dependent cyton model and that this form of immune regulation permits rapid induction of a wide range of responses from tolerance to strong immunity on the basis of small changes in mean time to divide, die or differentiate (10, 20). There is very strong evidence that murine lymphocytes *in vitro* obey the cyton model (10, 11, 20, 21, 24). To investigate whether T lymphocytes in humans *in* vivo also obey this age-dependent model, we combined mathematical modeling, analysis of existing heavy water labeling data and generation of new Annexin V and deuterated glucose labeling data.

Our main conclusions are that firstly, the choice of model paradigm has a large impact on estimates of cell lifespan obtained using stable isotope labeling. It seems likely that this conclusion will also apply to the interpretation of CFSE and BrdU labeling data. Secondly, we found no evidence to support the cyton model as a description of T cell dynamics in humans *in vivo*. No version of the cyton model (neither homogeneous nor heterogeneous, lognormal nor gamma) ever provided the best description of *in vivo* heavy water labeling. This was true for all four T cell populations considered: naïve CD4^+^ T cells, naïve CD8^+^ T cells, memory CD4^+^ T cells and memory CD8^+^ T cells. An alternative approach which was independent of model complexity (fitting an unconstrained gamma or lognormal distribution) also found no evidence for the cyton model. Furthermore, the cyton model (all versions) also failed to predict patterns in an independent, newly generated data set. Thirdly, the age-independent exponential model provided the best description of both naïve and memory T cell dynamics. Age-independent models provided the best fit to naïve T cell labeling data in 10/10 cases and to memory T cell labeling data in 7/10 cases, though differences were not always large. The age-independent model was also able to predict the novel Annexin V dataset. Our finding in favor of an age-independent paradigm is not inconsistent with the Hayflick limit. Age as defined by telomere shortening is counted from haematopoiesis and is distinct from, and on a different timescale to, the age since division which we focus on. Additionally, the action of telomerase, which is up-regulated in dividing lymphocytes (47), may mean that most T cell death is independent of the Hayflick limit. Time since haematopoiesis will be related to the age of the individual subject, the association between age of the individual and T cell dynamics has recently been studied in mice (48).

Given that the evidence for the cyton model *in vitro* is so strong, it is surprising that it fails to explain lymphocyte dynamics *in vivo*. There are at least three possible explanations. Firstly, the difference may relate to the difference between conditions *in vitro* and *in vivo. In vivo*, even if a cell's intrinsic programme is age-dependent, randomly encountered cell-exogenous stimuli such as cytokines, cell-cell contact or antigen may render its resultant behavior age-independent. That is, lymphocytes may have an age but other factors dominate *in vivo* so in practice age does not determine their fate (49). Of note, in many of the *in vitro* experiments supporting the cyton model, autocrine Il-2 is blocked (20, 24, 50). Specifically, in cultures of murine T cells, murine IL-2 (mIL-2) is blocked by the addition of an antibody and then the media supplemented with a fixed concentration of human Il-2 which is active on murine T cells but not blocked by the mIL-2-specific antibody. In this way the natural effect of autocrine Il-2 is removed. This is one example of a possible stimuli that would be present *in vivo* but has been excluded *in vitro* and which could have a profound effect on T cell behavior. Secondly, the difference may relate to differences between mice and humans. Human and murine immune systems face different selection pressures and may have evolved different behavior. For instance, there is evidence that the lifespan of memory and naïve T cells and the maintenance of naïve T cell homeostasis are very different in humans and mice (15). However, a study by Gossel et al. (39) which studied the dynamics of memory CD4^+^ T cells in mice *in vivo*, also found that Ki67^+^ (“young”) cells had lifespans that were indistinguishable from Ki67^−^ (“old”) cells providing strong support for an age-independent model compared to age-independent alternatives in mice *in vivo*. This agrees well with our conclusions in humans, suggesting that the difference is more likely to relate to *in vitro* v *in vivo* conditions rather than mice v humans. Finally, the difference may relate to differences in stimulation. In the scenarios we modeled there was no overt infection, whilst in the work supporting the cyton model, lymphocytes were always stimulated exogenously. Since the individuals we studied did not have any symptomatic infection we did not simulate the “division destiny” of lymphocytes (duration of the division burst of B and T cells after stimulated *in vitro*) which is a feature of the cyton model (10, 24). It would be interesting to repeat our work in individuals with ongoing infection to see if the rules of cell fate were altered. Nevertheless, the authors of the cyton work have always argued that the cyton paradigm also explains lymphocytes in homeostasis (10) and have investigated the consequences of the cyton paradigm in homeostasis upon thymus involution and aging (51, 52).

A potential concern about our approach is the use of the AICc for comparing models. Although such an approach is an appropriate, accepted technique (40) and is widely used for model selection e.g., (53, 54) it does identify the minimal model needed to describe the data rather than the “true” model. For naïve T cells, all the models fitted the data in a very similar way and so the winning model is necessarily the model with the least number of parameters i.e., the age-independent homogeneous model. For this reason our conclusion for the naïve cells is simply that there is no evidence to support age-dependence. However, for memory T cells, age-independent models consistently out-perform age-dependent models despite not having consistent differences in complexity. The winning model in most cases (age-independent heterogeneous) is more complicated than the cyton model (3 parameters for age-independent compared with 2 for cyton). When data becomes sparse (some people missed some sampling time points) and there is insufficient data to support the heterogeneous age-independent model, the winning model jumps to the homogeneous age-independent model (1 parameter) rather than any of the age-dependent options which have intermediate complexity. These conclusions were supported by a number of additional pieces of work. Firstly, *in silico* simulations suggests that our approach is unlikely to incorrectly favor an age-independent model if the generating model is actually the cyton model; if anything the approach is biased against age-independence. Secondly, when using a model with the same number of parameters for the age-independent, cyton and risk model (i.e., gamma distribution with unconstrained parameters) then for memory cells the age-independent model was strongly favored. Finally, our conclusions are supported by predictions of a new independent data set.

All the models considered contain a number of simplifying assumptions. This is essential to avoid over parameterization but has the potential to affect our results. Firstly, we assume that there is no inheritance of times to die or to divide between subsequent generations of cells. To a first approximation this is true; even in the earlier studies of cell fate where detailed *in vitro* measurements were made there was no evidence to support inheritance (10, 20, 22, 55). However, very fine single cell fate mapping has revealed signals for complex inheritance between cousins (21). Secondly, we neglect cell flow into the cell populations. We have shown in humans (34) and others have shown in mice (39) that such constitutive flow is ongoing even in homeostasis. Finally, we have assumed that both death and division of a given population can be described by the same probability density function (same parameters). The impact of these various assumptions cannot be directly explored due to the number of additional, correlated parameters which would need to be introduced. For instance, to investigate the third point above, we relaxed the assumption that death and division were described by the same distribution and considered two additional homogeneous age-dependent models of cell fate, in which division and death mechanisms were expressed by different probability density functions of the same form (gamma or lognormal). These models never outperformed the other versions in any of the cases but this is most likely due to the excess complexity of the new models (i.e., the data are not sufficiently rich to support the more complex models). Although it is not possible to explicitly analyze these simplifying assumptions we note that they have been applied to all paradigms equally. Finally, all participants were aged under 35 years. We therefore cannot determine whether the rules of cell fate in older individuals are best described by a cell age-dependent or cell age independent model.

In general, the work that can be performed in humans is very limited compared to what can be achieved with murine cells *in vitro*. We are restricted to studying population averages rather than single cells and must rely on natural physiology rather than use knockouts or transfectants. Nevertheless, the different paradigms do predict distinct patterns of behavior that can be detected at the cell population level in the absence of manipulation enabling us to distinguish between them. In summary, the cyton model provides a compelling hypothesis to explain how the immune system interleaves lymphocyte fate to achieve a robust, diverse response; it is strongly supported by *in vitro* work using murine cells. Unexpectedly, using three different approaches, we find no evidence that it describes T cell fate in healthy humans *in vivo*. Additionally, we show that extreme care needs to be taken in selecting models for estimating lymphocyte dynamic parameters as the choice of the underlying paradigm can have a profound impact on the estimates derived. Given the strength of the evidence for the cyton model *in vitro* we suggest that age probably determines cell fate *in vitro* but *in vivo*, where cells receive a huge number of external stimuli from other cells, antigen and cytokines, extrinsic factors rather than age are more likely to determine cell fate. A useful analogy is human lifespan. Fundamentally, the time when a human dies depends on their age and, in the developed world in peacetime, this pattern is manifest and age is a major determinant of the probability of death. However, during wartime, death is largely a random event and time of death is independent of a person's age. We suggest that the cyton model describes the fundamental rule of cell fate and is manifest “in peacetime” (*in vitro*) but in war (*in vivo*) these effects are overridden by the large number of cell-exogenous stimuli.

The age-independent exponential distribution is usually assumed but rarely justified. Here, we show that, *in vivo* for memory and naïve CD4^+^ and CD8^+^ T cells in healthy humans, the age-independent model appears to provide the best description of cell fate in homeostasis.

## Materials and Methods

### Agent-Based Model

We developed an agent-based model (ABM) to simulate stable isotope labeling of a population of cells in homeostasis. Upon birth (by division of the mother cell), cells were assigned times to die/differentiate and to divide which were drawn from a defined distribution (gamma or lognormal). The time step in the model is 1 day. After each time step, the age of the cell is increased by 1 day. If the age becomes equal to the time to die/differentiate, the cell is removed from the population. If it reaches its time to divide, it is substituted by two new daughter cells of age 0 with times to die/differentiate and to divide which are drawn again from the defined distribution. The model was implemented in C++.

#### Kinetic Heterogeneity

We allow for both kinetically homogeneous and heterogeneous populations. In a heterogeneous configuration, each subpopulation is assigned a different distribution of times to die/differentiate and times to divide. For a population consisting of n subpopulations, n-1 ratios are required to specify the proportions at which each of them is found. Each subpopulation is assumed to be independently in homeostasis, and the progeny of a given cell belong to the same subpopulation as the mother cell (i.e., its times to an event are drawn from the same distribution).

#### Deuterium Labeling Process

We simulate both heavy water and deuterated glucose labeling experiments. During the labeling period τ, each time that a cell divides, it is replaced by two daughter cells that are labeled in proportion to the label availability. In the case of heavy water, the availability of label is described by the following empirical equation (32):

$$\begin{array}{l} \;U\left(t\right)=f\left(1-e^{-\delta t}\right)+\beta e^{-\delta t}\;\text{during label intake }\tau \leq t\\ U\left(t\right)=\left[f\left(1-e^{-\delta \tau }\right)+\beta e^{-\delta \tau }\right]e^{-\delta \left(t-\tau \right)}\text{ after label intake }t>\tau \end{array}$$

In the case of deuterated glucose, because of the fast turnover rate of glucose, availability of label is assumed to follow a square pulse (17):

$$\begin{array}{l} U\left(t\right)=U\;\text{during label intake }t\leq \tau \\ U\left(t\right)=0\;\text{after label intake }t>\tau \end{array}$$

The parameters (*f*, δ, β*, U*) were estimated by fitting to successive measurements of label availability in plasma in the case of deuterated glucose or urine in the case of heavy water (Supplementary Table S4).

#### Model Initialization

In the ABM the remaining lifetime of the population will evolve in time as the population ages. Since we were studying adults, we assumed the cell population had reached steady state. We therefore initialize the times to die/differentiate and to divide in the ABM, from the steady state distributions of remaining lifetime which we derived analytically (Supplementary Information).

#### Fitting the Model

The fitting of the ABM to the experimental data with an ordinary least squares objective function was carried out using the global optimization algorithm pseudoOptim from the FME package in R (56). To minimize stochasticity, 5 different random seeds were considered each time a labeling curve was generated in C++ and the median of the 5 costs used. The number of free parameters depended on the model assumed (Table 1).

#### Age-Independent, Cyton, and Risk Paradigms

The three paradigms are captured by non-overlapping parameter spaces of the gammas and lognormal distributions of times to die/differentiate and to divide. The parameter ranges of the fitting procedures were configured accordingly for each case. For the gamma distribution, shape parameters equal to one (exponential distribution) are characteristic of an age-independent population; shape parameters lower than one (zero mode) are characteristic of the risk model; and shape parameters higher than one (higher than zero mode) are representative of the cyton model. For the lognormal distribution, we imposed the restriction that distribution modes had to be higher than 7 days, or lower than 2, respectively. The upper bound on the scale of the lognormal was set to 2.5; this was sufficient to observe dramatic risk behavior. Increasing the scale parameter to a value of 3 improved the fits of the homogeneous age-dependent risk model with one lognormal (not enough to change the results), but resulted in lifetimes which were unrealistic (in many cases, more than 25% of the population died or divided in the first 24 h. after division), Supplementary Table S3. Extending these limits further resulted in unfeasible fitting times due to the integration routines in the ABM. Additional details regarding the ABM are included in the Supplementary Information. Examples of the relationship between the distributions of time to die/differentiate and divide and the age-dependence of probabilities are provided in Supplementary Figure S1.

### Corrected Akaike Information Criterion (AICc)

We use the AICc metric to compare different models (40). The AICc introduces a penalty for the number of parameters and a correction for small sample size:

$$\begin{array}{l} AICc=n\ln\;\left[\frac{ssr}{n}\right]+\frac{2nk}{n-k-1} \end{array}$$

where *ssr* is the sum of squared residuals, *k* is the number of estimated parameters and *n* is the number of data points. The best model is identified by the lowest AICc. A frequently used rule of thumb states that an AICc difference <2 is not sufficient to empirically support the winning model; that a difference of 6 is considered as sufficient; and that a difference >10 favors one model with confidence (40). The absolute value of the AICc is not meaningful so we report the normalized AICc which is defined as the difference between the AICc and the AICc of the winning model (and is therefore zero for the winning model).

### Approach Validation

To investigate whether the approach of fitting the different models to the data and selecting the model with the lowest AICs yielded the correct model (i.e., the model which generated the data) we performed the following study. For each of the six models considered (homogeneous exponential, heterogeneous exponential, homogeneous gamma cyton, homogeneous gamma risk, homogeneous lognormal risk and homogeneous lognormal cyton) we simulated data. Specifically, we first simulated data from the model using realistic parameters (parameters were chosen based on fits to the heavy water data from the previous section), added noise to each data point [random variables sampled from N(0, 0.025)] and selected datasets of varying size (*n* = 6, *n* = 11, *n* = 20, *n* = 30, *n* = 50). For the *n* = 11 simulated data we chose timepoints which coincided exactly with our experimental time points; *n* = 6 is a subset of these and *n* > 11 an extension of these. We then fitted each of the 6 models to each of these 5 × 6 = 30 datasets and calculated the AICc. We then analyzed whether the winning model (model with the lowest AICc) was the model which was known to generate the data.

### Alternative Approach

We also developed an approach that did not rely on the AICc and was thus independent of model complexity. Instead of constraining the gamma or lognormal distributions to adopt parameters consistent with a given paradigm and comparing the fits we allowed the distributions to take up any parameter combination, effectively allowing the distributions to vary across the paradigms. For the gamma distribution there is a clear mapping between the parameters of the distribution and the paradigms (shape parameter = 1: age-independent distribution, shape parameter <1: risk paradigm, shape parameter > 1: cyton paradigm). The lognormal distribution is less useful as it cannot capture the age-independent paradigm and the mapping between parameters and age-dependent paradigms is imprecise. To investigate the best fitting lognormal distributions we plotted the probability of an event conditioned on survival to that point calculated from the best fit distribution (equations given in Supplementary Information). For the risk paradigm the distribution of the conditional probability would be expected to be strongly left-skewed with a decreasing probability with age, for the cyton paradigm there would be more weight to the right with probabilities increasing with age. To illustrate the spread of distributions we also plotted 100 trajectories created by sampling the parameters from the normal distribution centered on the best fit parameter with standard deviation the standard deviation of the parameter estimate.

### Heavy Water Data

Previously published *in vivo* heavy water labeling data from five subjects were re-analyzed using the ABM (above). Experimental details have been published elsewhere (32). Briefly, healthy young adults (age 20–25) received heavy water for 9 weeks comprising a prime of 10 ml ^2^H_2_O/kg body weight over 24 h followed by 1.25 ml ^2^H_2_O/kg body weight daily. Cell samples were taken at successive time points; urine samples were measured for body water enrichment. Label enrichment in the DNA of Naïve (CD45RO^−^CD27^+^) and Memory (CD45RO^+^), CD4^+^ and CD8^+^ T cells were measured by GC-MS.

### Deuterated Glucose and Annexin V+ Data

Four healthy adults (independent of the individuals studied by heavy water labeling) were studied (age 21–33, median 24.5). Briefly, all received 6,6-^2^H_2_-glucose as a primed, 24 h intravenous infusion. Cells dividing during the labeling phase become labeled by incorporation of deuterium into their DNA. Fifty milliliter of blood was taken at days 3, 4, 7, and 8; PBMC were isolated by Ficoll gradient centrifugation and CD3^+^CD4^+^CD45R0^+^ lymphocytes were separated within 4 h of venepuncture by flow cytometry (Moflo flow cytometer, Cytomation, Fort Collins, CO) according to Annexin V binding into two populations: CD4^+^CD45RO^+^ Annexin V-bright and CD4^+^CD45RO^+^ Annexin V-negative T cells; cells expressing intermediate levels of Annexin-staining were not collected in this analysis. Forward/side-scatter gating was used to exclude dead cells and cellular debris. DNA was extracted and hydrolysed to nucleosides and deuterium enrichment in DNA measured by gas-chromatography mass-spectrometry as previously described (5, 12) using the PCI derivative monitoring ions m/z 198 and 200 (Agilent GCMS, Agilent, Bracknell, UK).

The fractions of cells labeled and unlabeled at each time point were quantified for both Annexin V-positive and –negative cell populations for the 4 subjects. The fraction of Annexin V-positive cells amongst labeled and unlabeled cells were derived from those fractions as the ratio of the Annexin V-positive cells and the sum of both Annexin V-positive and -negative populations in labeled and unlabeled groups, respectively. This cannot be measured directly i.e., we cannot sort labeled and unlabeled cells and then quantify Annexin V expression as the measurement of label requires the destruction of the cells (their DNA is isolated and hydrolyzed).

## Data Availability Statement

The datasets generated for this study are available on request to the corresponding author.

## Ethics Statement

The studies involving human participants were reviewed and approved by NRES Committee London—Chelsea (Ref 10/H0803/102 and 13/LO/0022). The patients/participants provided their written informed consent to participate in this study. All interventions were performed according to the principles of the Declaration of Helsinki.

## Author Contributions

PC designed research, performed research, analyzed data, and wrote the paper. BD contributed vital new computational tools and analyzed data. MR-M performed research. SN contributed vital new computational tools. AW, DW, and PB performed research. DM performed research and wrote the paper. BA designed research, performed research, analyzed data, and wrote the paper.

### Conflict of Interest

The authors declare that the research was conducted in the absence of any commercial or financial relationships that could be construed as a potential conflict of interest.
