# Supplementary material for: The Rules of Human T Cell Fate in vivo
Source: Front Immunol. 2020 Apr 8;11:573. doi: 10.3389/fimmu.2020.00573 (PMC7156550; doi:10.3389/fimmu.2020.00573)
Supplement: Supplementary file 1 [file Data_Sheet_1.docx]

**SUPPLEMENTARY INFORMATION**

Contents

[Supplementary Methods 2](#_Toc36206413)

[Derivation of the pdf of remaining lifetime at equilibrium 2](#_Toc36206414)

[Further details of the agent-based model 4](#_Toc36206415)

[Supplementary Figures 6](#_Toc36206416)

[Supplementary Figure S1. Examples of lifespan distributions and conditional probability for age-independent and age-dependent paradigms. 6](#_Toc36206417)

[Supplementary Figure S2. Best fit distributions for naive T cells. 8](#_Toc36206418)

[Supplementary Figure S3. Best fit distributions for memory T cells. 9](#_Toc36206419)

[Supplementary Figure S4. Best fit of the 6 models to Naïve CD4+ and CD8+ T cell data. 10](#_Toc36206420)

[Supplementary Figure S5. Alternative approach to determining paradigm 13](#_Toc36206421)

[Supplementary Tables 14](#_Toc36206422)

[Supplementary Table S1. Normalised AICc for fits to naïve T cell data. 14](#_Toc36206423)

[Supplementary Table S2. Normalised AICc for fits to memory T cell data. 14](#_Toc36206424)

[Supplementary Table S3. Parameter constraints for model fitting. 15](#_Toc36206425)

[Supplementary Table S4. Parameters of Label Availability. 15](#_Toc36206426)

# Supplementary Methods

## Derivation of the pdf of remaining lifetime at equilibrium


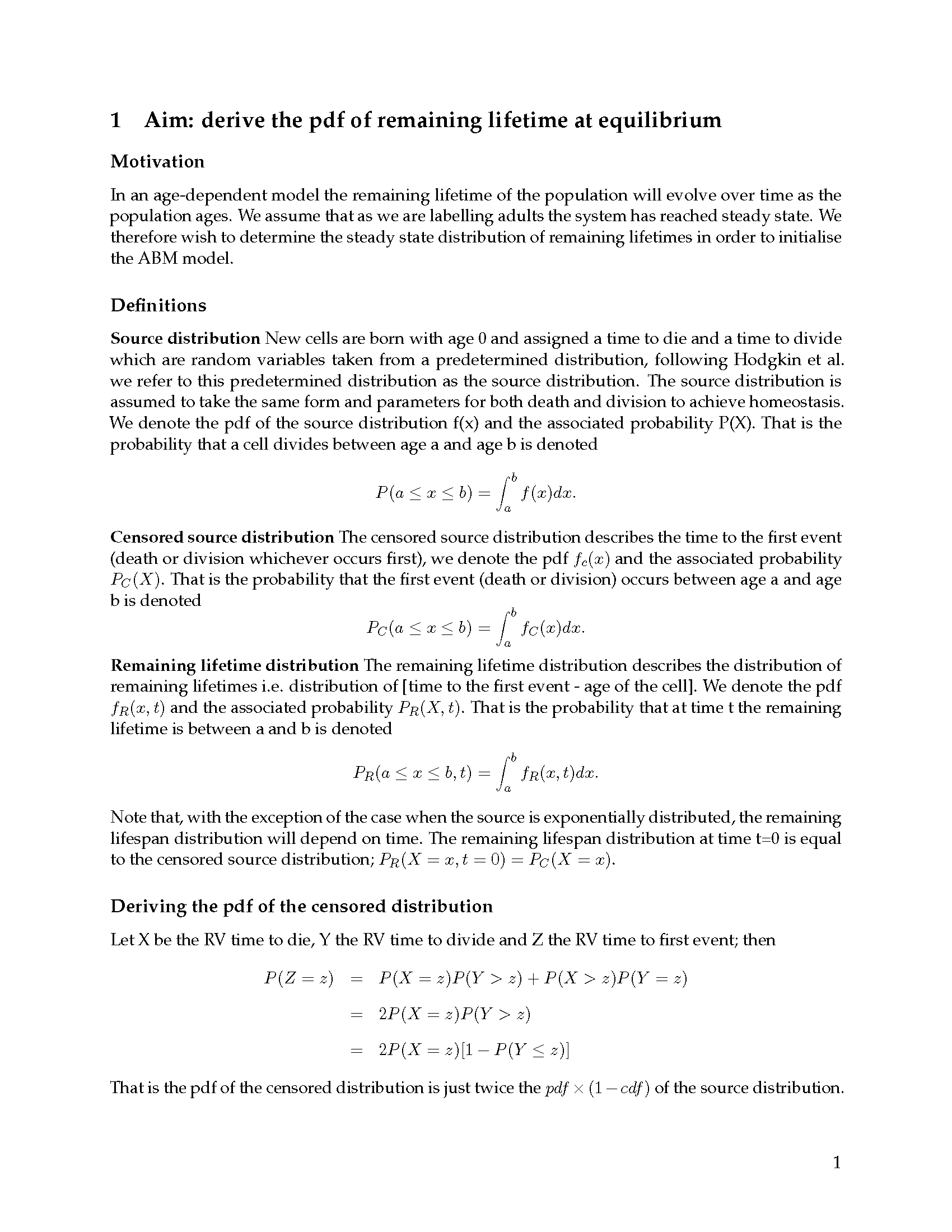


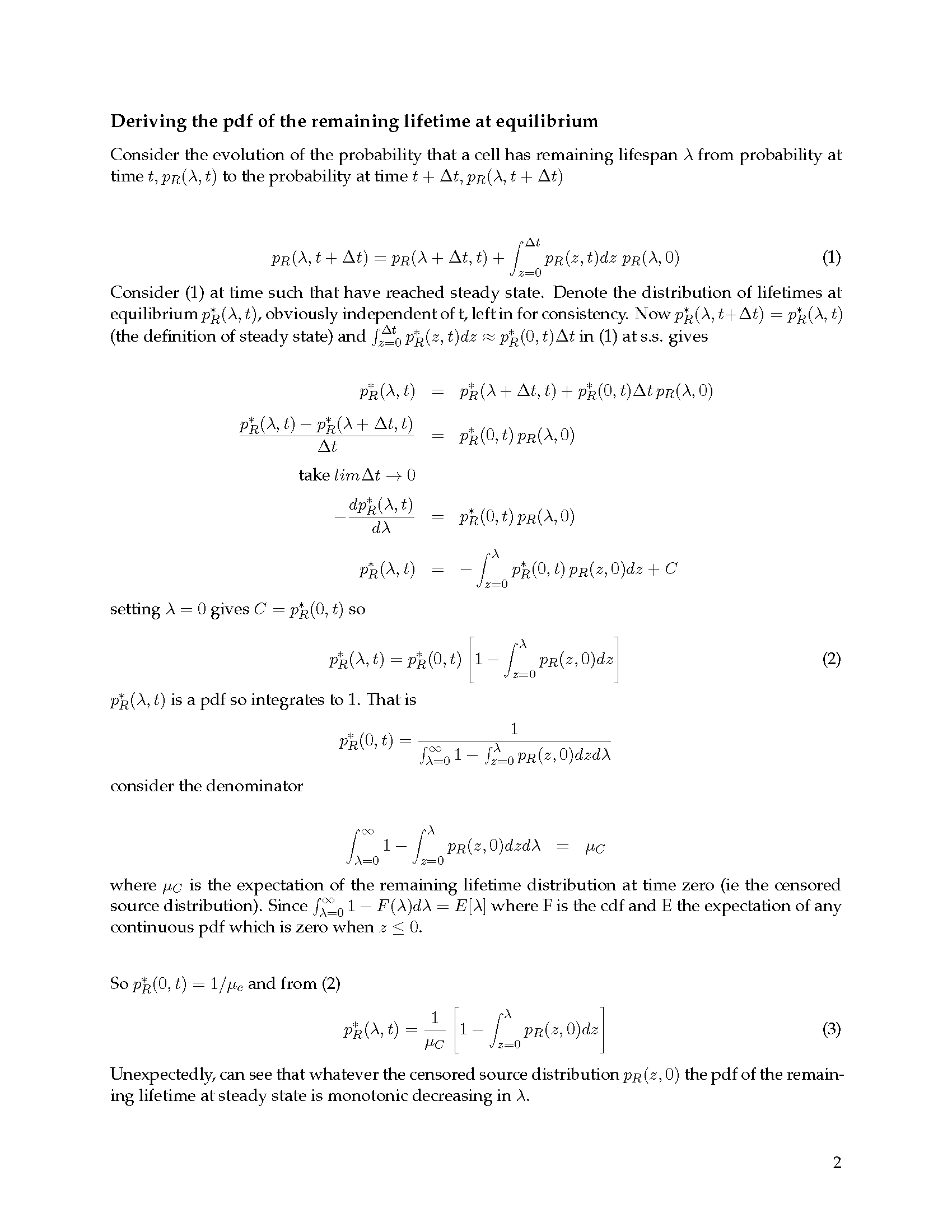


## Further details of the agent-based model

The proposed agent-based model (ABM) represents death and division events by sampling times to die or to divide drawn from a gamma or lognormal distribution. This sampling scheme offers a flexible way to simulate stochastic cell population dynamics and is related to several existing quantitative models for T cell population dynamics. Here, we discuss these relations.

***Equivalence of the ABM to deterministic models & the Gillespie algorithm***

In general, stochastic birth-death Poisson processes for a sufficiently large population size would be expected to exhibit equivalent population dynamics to that generated by a deterministic equation [S1]. We have confirmed that the homogeneous age-independent model, as a special case of the ABM, is technically equivalent to the conventional Gillespie algorithm. Additionally, we demonstrated that the homogeneous age-independent version of the ABM can fit to the heavy water labelling data of several cell types essentially the same as the deterministic ordinary differential equation formulation of an age-independent process (**Figure 3** and data not shown).

***Relationship of the ABM to the cyton model***

In [S2], it is shown that several existing mathematical models that describe T and B cell population dynamics measured by CFSE labeling can be reformulated as a system of integral equations. The cyton model is included as a special case of their proposed model. In [S3] it is demonstrated that the sampling scheme employed in the ABM can produce a stochastic representation of the population dynamics exhibited by the integral equations of [S2]. In other words, our ABM can formally reproduce population dynamics of the cyton model as stochastic sample paths when distributions for times to die and to divide are chosen to be those assumed in the cyton model.

***A mathematical condition that ensures maintenance of steady state***

Experimental data were taken from adults in which the cell population has reached a steady state. We therefore constrained the distributions of times to die and to divide in the ABM to maintain a constant population size. In [S3], a generation progression ratio was introduced to quantitatively describe the ratio of population sizes between two successive generations. The population size remains constant if generation progression ratios between any of two successive generations are equal to one. This condition is satisfied if times to die and to divide are taken from identical distributions (i.e. same distribution, same parameters). We also relaxed this assumption by allowing times to divide and die to be drawn from the same family of distributions but with different parameters governing the two distributions. In this case steady state was maintained by constraining the expected values of both distributions to be the same. We show numerically that this is sufficient to maintain steady state.

***Determination of feasible initial population size in agent-based simulations***

The ABM produces stochastic sample paths due to the finite number of individuals in the population. This stochasticity may affect the quality of fitting. To determine feasible population sizes that produce stable fits the homogeneous age-independent model with different initial population sizes was fitted to *in vivo* deuterium water labeling data of granulocytes and T cells. Stable estimates were obtained when initial population sizes are greater than 1000 (results not shown). We chose a population size of 10,000. To further minimize the effect of the stochasticity on those estimates, the median cost of five labelling curves generated with five different seeds was considered in each iteration of the fitting process. It should be noticed that we do not use an ABM to simulate the stochasticity of the system but because it provides a flexible way to incorporate different cell fate models; a deterministic approach such as partial differential equations would be another option.

**References for Supplementary Information**

[S1] D.T. Gillespie, Stochastic Simulation of Chemical Kinetics, Annual Review of Physical Chemistry 58 (2007) 35--55.

[S2] A. Zilman, V. V. Ganusov and A. S. Perelson, Stochastic models of lymphocyte proliferation and death, PLoS One, 5 (2010), e12775.

[S3] S. Nakaoka and H. Inaba, Demographic modeling of transient amplifying cell population growth, Mathematical Biosciences and Engineering, 11(2) (2014) 363-384.

# Supplementary Figures

Distribution of time to an event (death, differentiation, division)

Distribution of time to first event

Probability of event at a given age conditional on survival to that age

## **Supplementary Figure S1**. Examples of lifespan distributions and conditional probability for age-independent and age-dependent paradigms.

For each of the three paradigms: age-independent, risk and cyton (row 1, 2 and 3 respectively) the distribution of time to an event (death, differentiation and division, first column), distribution of time to any event (second column) and instantaneous probability of an event at a given age conditioned on survival to that age (third column) are plotted. In the Supplementary Information the distribution of time to an event is referred to as the source distribution and the distribution of time to any event is referred to as the censored source distribution. In all cases the underlying (source) distribution is the gamma distribution.

**A.** Probability distribution of the time to an event for an age-independent distribution (gamma with shape=1 and rate=0.01, equivalent to exponential with rate=0.01). **B.** Probability distribution of the time to the first event (death, differentiation or division) for an age-independent distribution (gamma with shape=1 and rate=0.01). **C.** Probability of an event occurring immediately after age on x axis given survival to that age for an age-independent distribution (gamma with shape=1 and rate=0.01). **D**, **E**, **F** as for **A**, **B**, **C** but for an age-dependent distribution consistent with the risk paradigm (gamma with shape=0.01 and rate=0.01). **G**, **H**, **I** as for **A**, **B**, **C** but for an age-dependent distribution consistent with the cyton paradigm (gamma with shape=10 and rate=0.01). Note the different scales of the x axis and y axis in the different panels.

It can be seen that for the age-independent model the probability of an event at age x (given survival to age x) is independent of age (**C**), for the risk model the probability is highest at birth and then decreases with age (**F**) and for the cyton model the probability is initially zero and increases with age (**I**).

The functions plotted are as follows

Probability of an event at age $x$: $P(x)=\frac{1}{\theta^{\alpha}\Gamma(\alpha)}x^{\alpha-1}e^{-x/\theta}$

Probability of any event (death/differentiation or division) at age $x$: $P(x)=2\frac{1}{\theta^{\alpha}\Gamma(\alpha)}x^{\alpha-1}e^{-x/\theta}\int_{y=x}^{\infty} \frac{1}{\theta^{\alpha}\Gamma(\alpha)}y^{\alpha-1}e^{-y/\theta}dy$

Probability of any event (death/ differentiation or division) at age $x$given survival to age $x:$ $P(x)=2\frac{1}{\theta^{\alpha}\Gamma(\alpha)}x^{\alpha-1}e^{-x/\theta}\int_{y=x}^{\infty} \frac{1}{\theta^{\alpha}\Gamma(\alpha)}y^{\alpha-1}e^{-y/\theta}dy \div\left[\int_{z=x}^{\infty} \left(2\frac{1}{\theta^{\alpha}\Gamma(\alpha)}z^{\alpha-1}e^{-z/\theta}\int_{y=x}^{\infty} \frac{1}{\theta^{\alpha}\Gamma(\alpha)}y^{\alpha-1}e^{-y/\theta}dy\right)dz\right]$

| Naïve CD4^+^ T cells |  |  |
| --- | --- | --- |
| 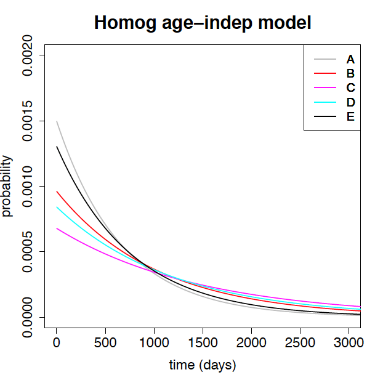 | 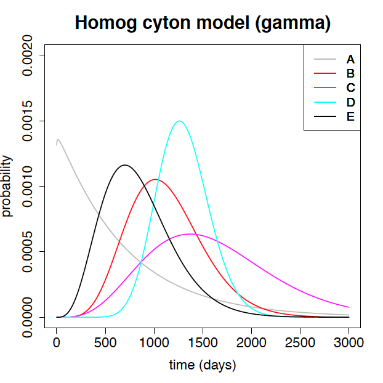 | 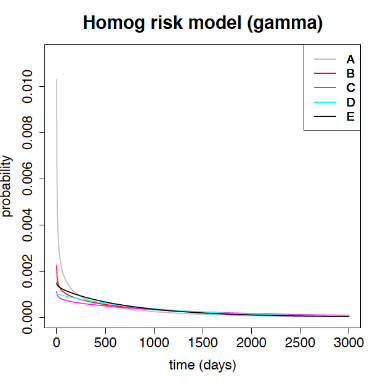 |
| Naïve CD8^+^ T cells |  |  |
| 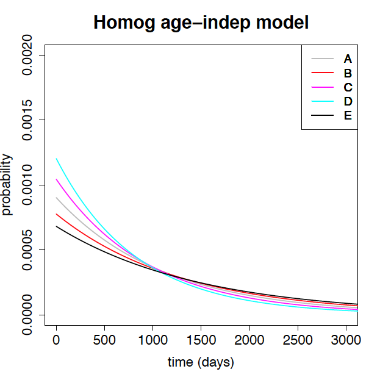 | 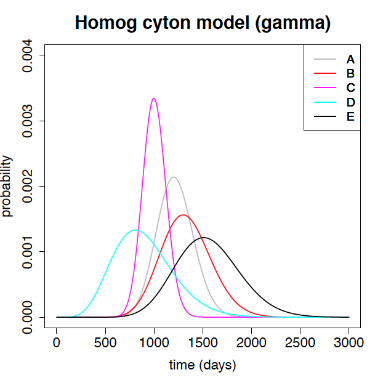 | 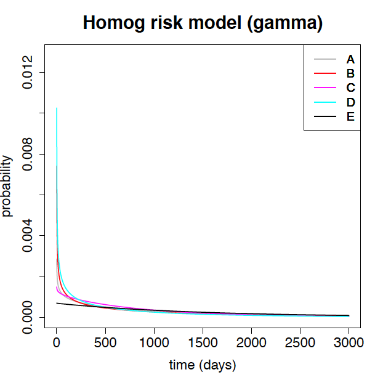 |

## **Supplementary Figure S2**. Best fit distributions for naive T cells.

The experimental data from 5 individuals (A-E) from the heavy water study were fitted with the homogeneous version of the three paradigms (age-independent, cyton and risk). The best fit censored lifespan distributions (i.e. the distribution of the time from cell birth by division of the mother cell to either death or division whichever occurs first) are plotted. Top row shows the best fits for naive CD4^+^ T cells and bottom row the best fits for naive CD8^+^ T cells. Each coloured curve corresponds to a different individual**.**

| Memory CD4^+^ T cells | |  |
| --- | --- | --- |
| 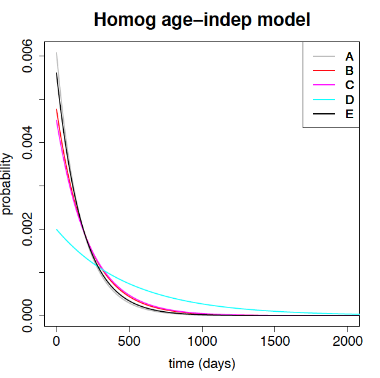 | 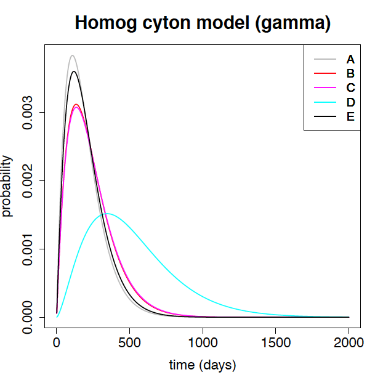 | 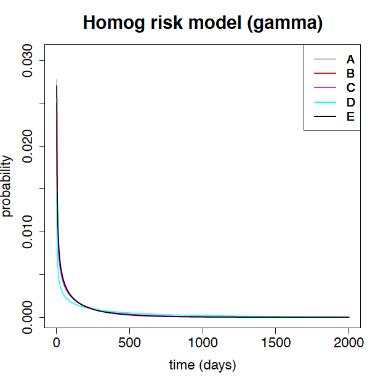 |
| 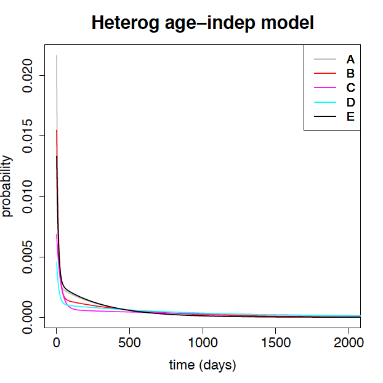 | 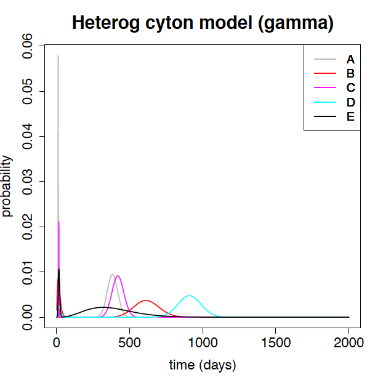 |  |
| Memory CD8^+^ T cells | |  |
| 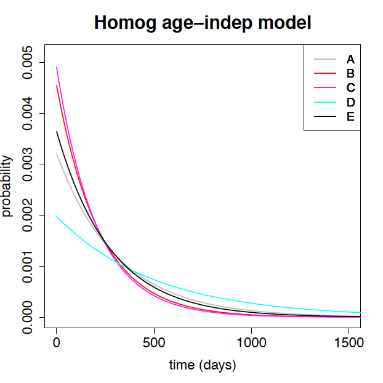 | 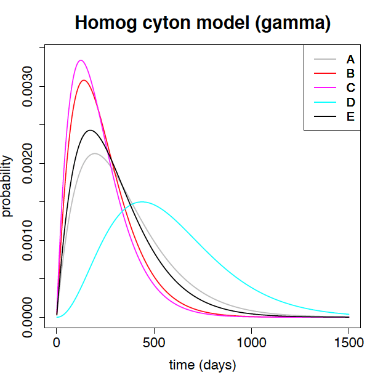 | 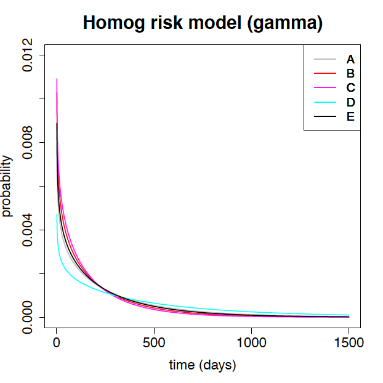 |
| 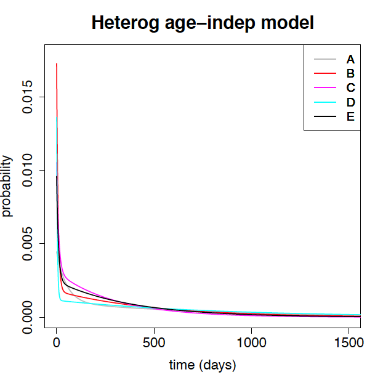 | 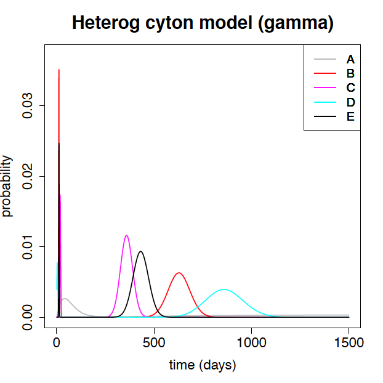 | 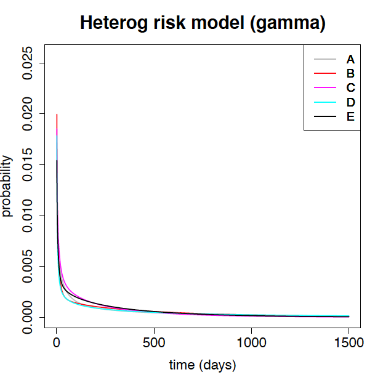 |

## **Supplementary Figure S3.** Best fit distributions for memory T cells.

The experimental data from 5 individuals (A-E) from the heavy water study were fitted with the homogeneous and heterogeneous versions of the three paradigms (age-independent, cyton and risk). The best fit censored lifespan distributions (i.e. the distribution of the time from cell birth to either death or division whichever occurs first) are plotted. Top two rows show the best fits for memory CD4^+^ T cells and the bottom two rows the best fit for memory CD8^+^ T cells. Each coloured curve corresponds to a different individual**.**

## Supplementary Figure S4. Best fit of the 6 models to Naïve CD4+ and CD8+ T cell data.

A different subject (A-E) is shown in each panel. Here the cyton and risk models are described using the gamma distribution; similar results are obtained using the lognormal distribution for the cyton model whilst the lognormal version of the risk model provided a very poor fit to the data. Data taken from [33].

| **A. Homogenous lognormal** | | | | |
| --- | --- | --- | --- | --- |
|  | Naïve CD4^+^ T cells | | Naïve CD8^+^ T cells | |
| A  B  C  D  E | Probability → | 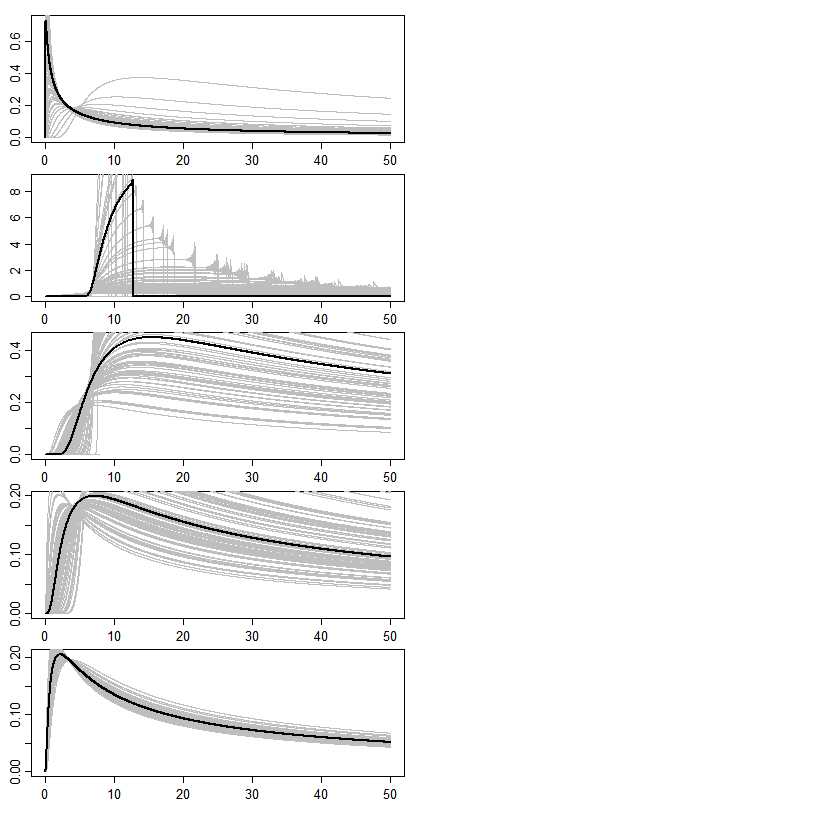 | Probability → | 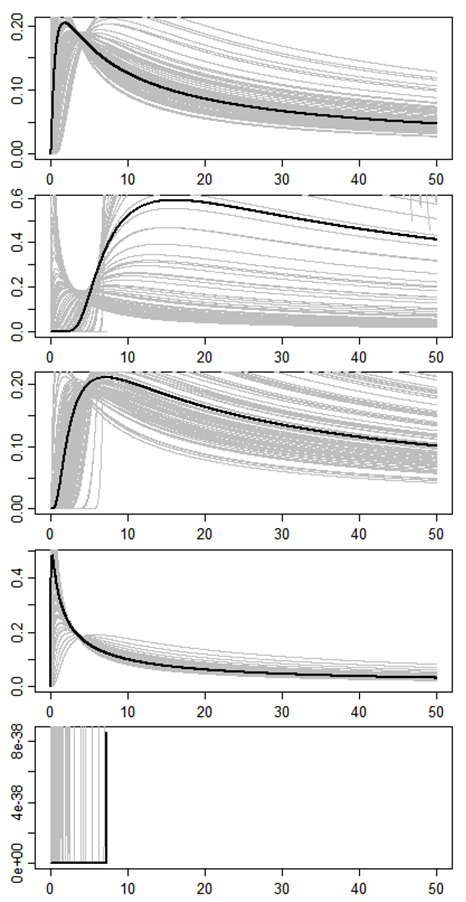 |
|  |  | Time (days) → |  | Time (days) → |
|  |  |  |  |  |
|  | Memory CD4^+^ T cells | | Memory CD8^+^ T cells | |
| A  B  C  D  E | Probability → | 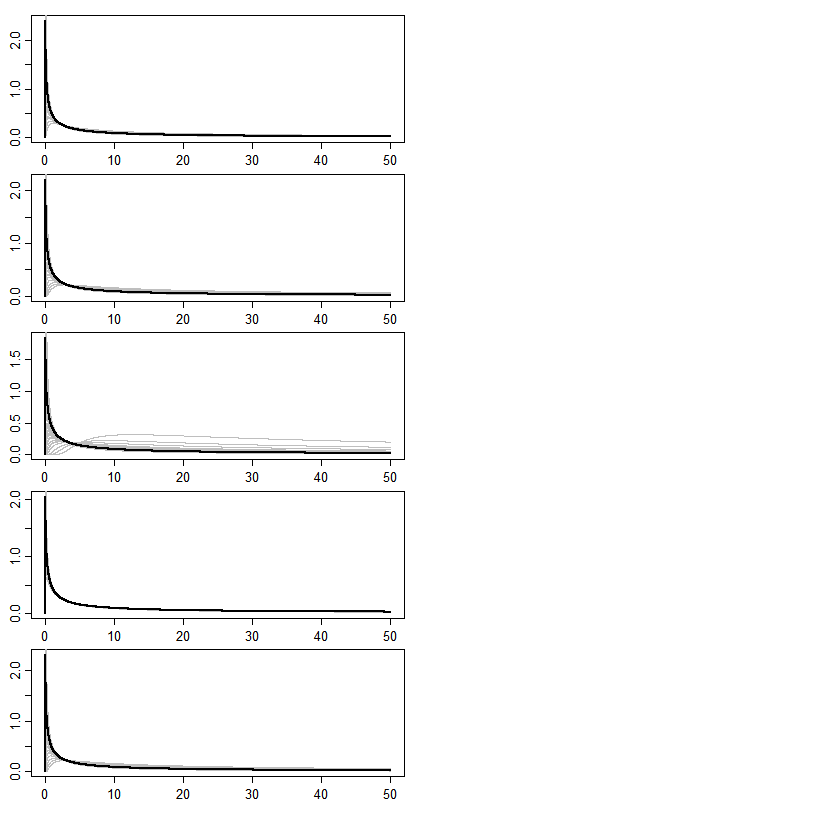 | Probability → | 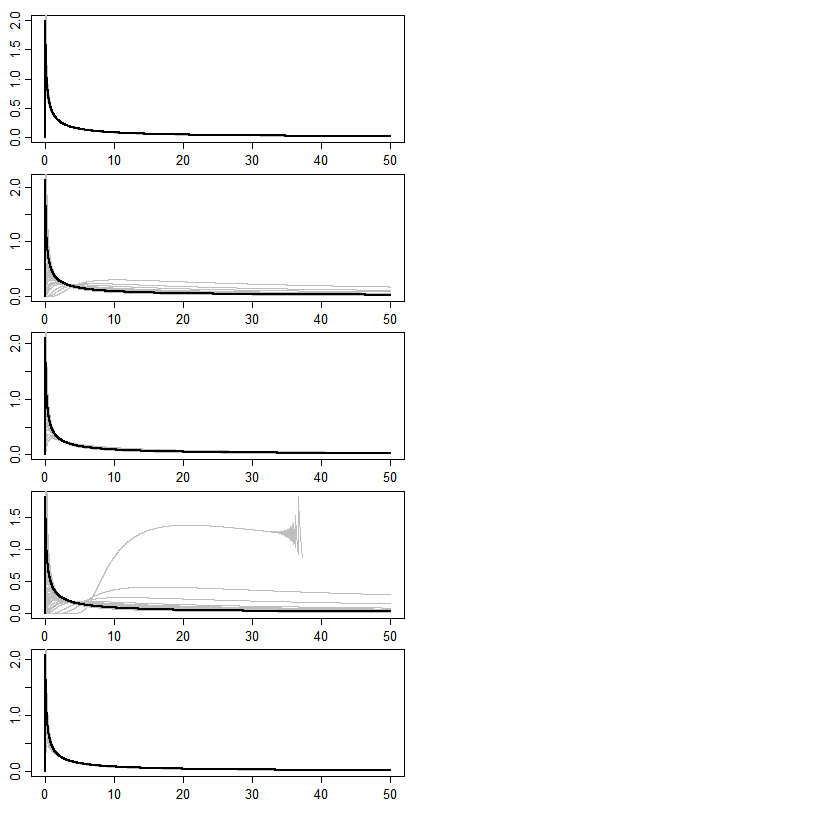 |
|  |  | Time (days) → |  | Time (days) → |
|  |  |  |  |  |

| **B. Heterogeneous lognormal** | | |
| --- | --- | --- |
|  | Memory CD4^+^ T cells | |
|  |  | Subpopulation 1 Subpopulation 2 |
| A  B  C  D  E | Probability → | 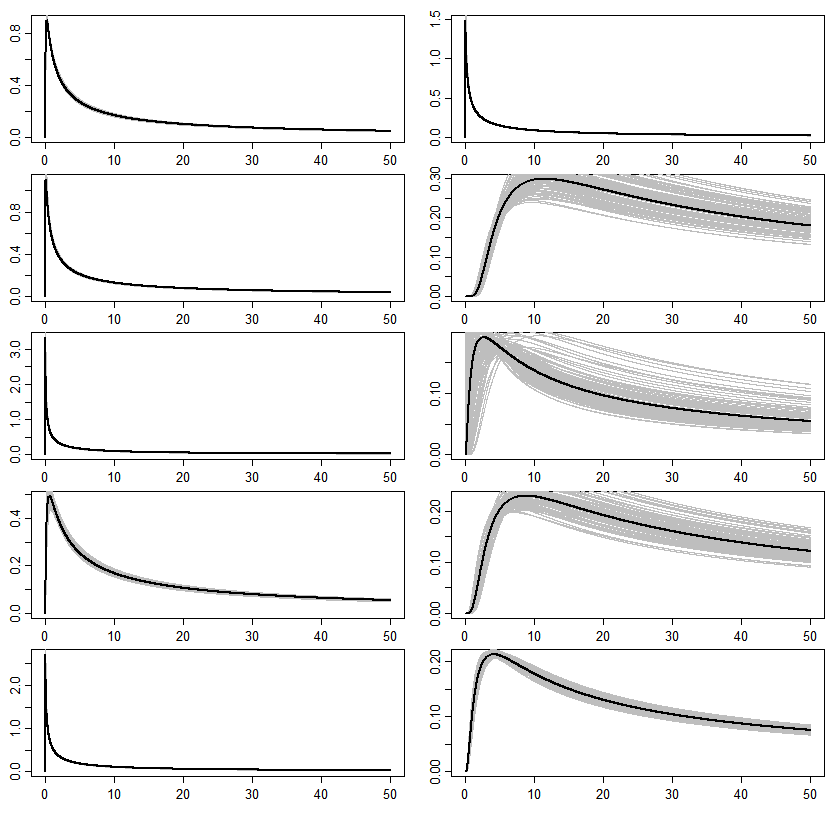 |
|  |  | Time (days) → |
|  |  |  |
|  | Memory CD8^+^ T cells | |
|  |  | Subpopulation 1 Subpopulation 2 |
| A  B  C  D  E | Probability → | 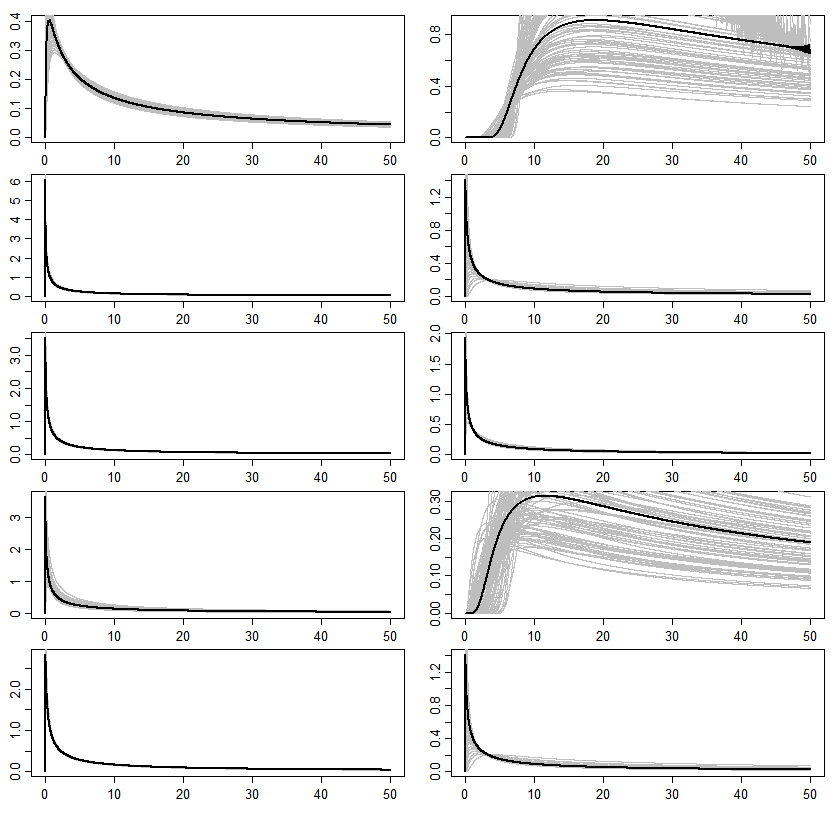 |
|  |  | Time (days) → |

## Supplementary Figure S5. Alternative approach to determining paradigm

Models based on lognormal distributions in which parameters were free to vary across both risk and cyton paradigms were fitted to the naïve CD4^+^, naïve CD8^+^, memory CD4^+^ and memory CD8^+^ T cell heavy water labelling data. The best fit distributions were found and the probability of an event conditional on survival to that point plotted. The thicker black line is the probability from the best fit distribution, the grey lines are 100 trajectories created by sampling the parameters from the normal distribution centered on the best fit parameter with standard deviation given by the standard deviation of the parameter estimate.

**A.** Probability distributions resulting from fitting homogenous lognormal model to naïve T cells (top panel) and memory T cells (bottom panel). CD4^+^ T cells are shown on left, CD8^+^ T cells on right.

**B.** Probability distributions resulting from fitting heterogeneous lognormal model to memory CD4^+^ T cells (top panel) and memory CD8^+^ T cells (bottom panel). Distribution for the faster subpopulation shown on the left, and for the slower subpopulation on the right.

The lognormal distribution cannot describe the age-independent paradigm so here we can only compare between the risk and cyton paradigms. For naïve T cells the distribution is rather poorly identified. For memory T cells the best fit distributions are strongly supportive of the risk paradigm (heavily left skewed with probability decreasing with age) rather than the cyton paradigm, particularly for the homogeneous model and for the faster of the two subpopulations for the heterogeneous model (the subpopulation that can best be identified from the labelling data).

# Supplementary Tables

|  |  | **Homogeneous** | | | | | **Heterogeneous** | | | | |
| --- | --- | --- | --- | --- | --- | --- | --- | --- | --- | --- | --- |
|  |  |  | **Gamma** | | **Lognormal** | |  | **Gamma** | | **Lognormal** | |
|  |  | **Age-indep** | **Cyton** | **Risk** | **Cyton** | **Risk** | **Age-indep** | **Cyton** | **Risk** | **Cyton** | **Risk** |
| **Naive CD4^+^ T cells** | A | **0** | 3.55 | 2.53 | 2.4 | 20.28 | 4.81 | 16.04 | 16.81 | 16.47 | 36.28 |
|  | B | **0** | 2.69 | 0.92 | 2.72 | 61.39 | 4.94 | 19.26 | 19.67 | 19.71 | 81.56 |
|  | C | **0** | 3.55 | 4.38 | 3.36 | 40.44 | 8.88 | 26.91 | 27.6 | 26.66 | 64.23 |
|  | D | **0** | 2.28 | 2.88 | 2.25 | 63.73 | 7.12 | 17.1 | 18.14 | 17.17 | 79.37 |
|  | E | **0** | 2.52 | 1.4 | 2.21 | 57.52 | 5.86 | 19.72 | 18.53 | 18.92 | 74.97 |
| **Naive CD8^+^ T cells** | A | **0** | 37.17 | 37.09 | 37.11 | 74.31 | 42.28 | 59.64 | 60.29 | 60.09 | 98.01 |
|  | B | **0** | 2.96 | 3.59 | 2.91 | 42.5 | 7.71 | 22.39 | 22.92 | 22.24 | 62.44 |
|  | C | **0** | 12.99 | 12.94 | 12.97 | 27.57 | 19.25 | 42.24 | 42.73 | 42.82 | 57.65 |
|  | D | **0** | 3.51 | 2.46 | 2.75 | 35.68 | 3.68 | 15.22 | 16.27 | 15.04 | 51.49 |
|  | E | **0** | 2.03 | 2.45 | 2.07 | 70.2 | 7.21 | 18.57 | 18.99 | 18.78 | 87.7 |

## **Supplementary Table S1**. Normalised AICc for fits to naïve T cell data.

Normalised AICcs for the fits of the ABM to 63 day heavy water labelling data in naïve CD4^+^ and CD8^+^ T cells. Bold font with grey shading denotes the winning model for that data set (i.e. model with the lowest AICc). AICcs are normalised by subtracting the AICc of the winning model (so the winning model has an AICc of zero by definition). The median normalised AICc is plotted in **Figure 2**.

|  |  | **Homogeneous** | | | | | **Heterogeneous** | | | | |
| --- | --- | --- | --- | --- | --- | --- | --- | --- | --- | --- | --- |
|  |  |  | **Gamma** | | **Lognormal** | |  | **Gamma** | | **Lognormal** | |
|  |  | **Age-indep** | **Cyton** | **Risk** | **Cyton** | **Risk** | **Age-indep** | **Cyton** | **Risk** | **Cyton** | **Risk** |
| **Memory CD4^+^ T cells** | A | 29.8 | 35.54 | 31.85 | 29.99 | 21.95 | **0** | 18.2 | 17.42 | 16.12 | 16.96 |
|  | B | 26.89 | 32.65 | 29.29 | 27.36 | 21.41 | **0** | 18.51 | 17.09 | 17.37 | 18.88 |
|  | C | 0.69 | 7.2 | 4.16 | 3.3 | **0** | 2.1 | 26.61 | 27.24 | 27.07 | 27.5 |
|  | D | 11.85 | 16.65 | 12.99 | 10.83 | 10.06 | **0** | 12 | 12.63 | 11.67 | 24.94 |
|  | E | 24.34 | 30.87 | 24.84 | 21.72 | 10.34 | **0** | 12.97 | 12.38 | 12.73 | 13.65 |
| **Memory CD8^+^ T cells** | A | 5.87 | 12.55 | 8.82 | 4.59 | **0** | 3.88 | 22.84 | 22.65 | 21.86 | 22.86 |
|  | B | **0** | 6.49 | 5.49 | 4.41 | 2.44 | 1.38 | 77.18 | 77.26 | 77.32 | 75.6 |
|  | C | 8.19 | 15.48 | 11.46 | 6.96 | **0** | 1.09 | 19.96 | 21.07 | 20 | 20.96 |
|  | D | **0** | 7.54 | 6.59 | 5.35 | 5.15 | 6.74 | Inf | Inf | Inf | Inf |
|  | E | 25.04 | 30.85 | 27.59 | 22.82 | 15.44 | **0** | 16.55 | 15.76 | 15.25 | 13.23 |

## **Supplementary Table S2. Normalised AICc for fits to memory T cell data.**

Normalised AICcs for the fits of the ABM to 63 day heavy water labelling data in memory CD4^+^ and CD8^+^ T cells. Bold font with grey shading denotes the winning model for that data set (i.e. model with the lowest AICc). Inf denotes infinity (there are too many parameters in the model for the number of data points available). AICcs are normalised by subtracting the AICc of the winning model (so the winning model has an AICc of zero by definition). The median normalised AICc is plotted in **Figure 2**.

| **Paradigm** | **Shape** | **Scale** |
| --- | --- | --- |
| **Age-independent** | 1 | (0.5, 3000) |
| **Risk** | (0.6,0.99) | (0.5, 3000) |
| **Cyton** | (2,100) | (0.5, 3000) |

## **Supplementary Table S3**. Parameter constraints for model fitting.

For the gamma distribution there is an exact correspondence between the shape parameter and the underlying paradigm. Therefore, when fitting age-independent, cyton and risk models based on the gamma distribution the parameter constraints tabulated above were used. For the lognormal distribution the correspondence is less precise and so we fitted all models using a single parameter range ((0.5, 3000) ; (0,2.5)) but then discarded parameter combinations giving a modal lifespan>2 days for the risk paradigm and <7 days for the cyton paradigm.

| **Individual** | **U** |
| --- | --- |
| Subject 1 | 0.2684 |
| Subject 2 | 0.2936 |
| Subject 3 | 0.3526 |
| Subject 4 | 0.2907 |

**Supplementary Table S4. Parameters of Label Availability.**

Parameters *f, δ,* β which describe label availability in subjects A-E in the heavy water labelling experiment and parameter U which describes label availability in subjects 1-4 in the deuterated glucose labelling experiment were estimated from successive measurements of label availability in plasma in the case of deuterated glucose or urine in the case of heavy water. Parameters for heavy water were, as expected, identical to those previously reported for the same data set [33] and so are not repeated here, parameters for deuterated glucose are reported above.
